# The Tumorigenic Role of Circular RNA-MicroRNA Axis in Cancer

**DOI:** 10.3390/ijms24033050

**Published:** 2023-02-03

**Authors:** Woo Ryung Kim, Eun Gyung Park, Du Hyeong Lee, Yun Ju Lee, Woo Hyeon Bae, Heui-Soo Kim

**Affiliations:** 1 Department of Integrated Biological Sciences, Pusan National University, Busan 46241, Republic of Korea; 2 Institute of Systems Biology, Pusan National University, Busan 46241, Republic of Korea; 3 Department of Biological Sciences, College of Natural Sciences, Pusan National University, Busan 46241, Republic of Korea

**Keywords:** circular RNA, microRNA, microRNA derived from transposable element, cancer

## Abstract

Circular RNAs (circRNAs) are a class of endogenous RNAs that control gene expression at the transcriptional and post-transcriptional levels. Recent studies have increasingly demonstrated that circRNAs act as novel diagnostic biomarkers and promising therapeutic targets for numerous cancer types by interacting with other non-coding RNAs such as microRNAs (miRNAs). The miRNAs are presented as crucial risk factors and regulatory elements in cancer by regulating the expression of their target genes. Some miRNAs are derived from transposable elements (MDTEs) that can transfer their location to another region of the genome. Genetic interactions between miRNAs and circular RNAs can form complex regulatory networks with various carcinogenic processes that play critical roles in tumorigenesis and cancer progression. This review focuses on the biological regulation of the correlative axis among circular RNAs, miRNAs, and their target genes in various cancer types and suggests the biological importance of MDTEs interacting with oncogenic or tumor-suppressive circRNAs in tumor progression.

## 1. Introduction

Only 2% of transcribed RNAs in the genome can be translated into proteins, while the remaining 98%, called the non-coding RNAs (ncRNAs), cannot be translated [1]. Previous studies have shown that ncRNAs, including long non-coding RNAs (lncRNAs), small interfering RNAs (siRNAs), small nuclear RNAs (snRNAs), small nucleolar RNAs (snoRNAs), circular RNAs (circRNAs), piwi-interacting RNAs (piRNAs), rRNA (ribosomal RNA), tRNA and microRNAs (miRNAs), determine the fate of cells as crucial modulators of indispensable biological processes by adjusting their genetic expression at the transcriptional, post-transcriptional, and epigenetic levels [2,3]. The ncRNAs have a direct correlation with each target gene and systematically regulate the expression of target genes through mutual regulation [4,5]. In particular, numerous cancer studies have shown that circRNAs and miRNAs can induce oncogenic phenotypes related to diverse cancer hallmarks through a tightly controlled relationship [6,7].

The miRNAs are endogenously expressed small non-coding RNAs (<200 nucleotides) that are robust candidates for the disease-specific biomarkers with high tissue- or cell-specific expression characteristics [8,9,10]. The biogenesis mechanisms of miRNAs involve several cleavage steps in the nucleus and cytoplasm [11]. The synthesis of miRNAs starts with the transcription of DNA sequences including miRNA coding gene, intronic region and transposable elements, resulting in the formation of stem-loop structured RNAs, called primary miRNAs (pri-miRNAs) [12,13]. The catalytic complex composed of endonuclease, Drosha, and DiGeorge critical region 8 (DGCR8) transforms pri-miRNAs into precursor miRNAs (60–70 nt, pre-miRNAs), which are then exported to the cytoplasm through the exportin-5 protein [14,15]. The ribonuclease dicer forms mature miRNAs that can cause either messenger RNA (mRNA) degradation or translational repression via incorporation into ARGONAUTE 1 protein (AGO1) in the RNA-induced silencing complex (RISC) [16]. Processed miRNAs suppress the expression of target genes by complementarily binding to the recognition sites, called the “seed region”(5–8 nt) within the 3′ untranslated region (UTR) of messenger RNA (mRNA) at the post-transcriptional level [17,18]. The canonical epigenetic regulation of miRNA proceeds by interaction between miRNA and its target mRNA through a mechanism in which miRNA directly binds to the target mRNA and inhibits mRNA expression. Any change in the levels of certain miRNAs can affect the target gene expression and thus influence cell homeostasis [19,20]. Based on their functional roles in cancer development, there are two major types of miRNAs: oncogenic miRNA (onco-miR) and tumor-suppressive miRNA (tumor-suppressive miRs). These miRNAs form a regulatory axis under the control of oncogenic or tumor-suppressive circRNAs. From the oncogenetic perspective, circRNAs are closely related to multiple oncogenic biological pathways that lead to multiple types of cancer by competitively binding to onco-miRs or tumor-suppressive miRs [21,22,23]. 

Additionally, most miRNAs overlap with the introns and exons of coding genes and are less frequently derived from sequences of transposable elements that can relocate their position to the other regions of the genome [24,25]. Dysregulation of miRNA derived from transposable element (MDTE)s’ expression leads to the development of several types of cancer [26,27]. Even though the core role of miRNAs containing MDTEs is to perform post-transcriptional gene regulation, there is also an increasing interest in their biofunction as interactions with other non-coding RNAs in their regulatory network, which has refined our understanding of RNA biology.

**Figure 1:**
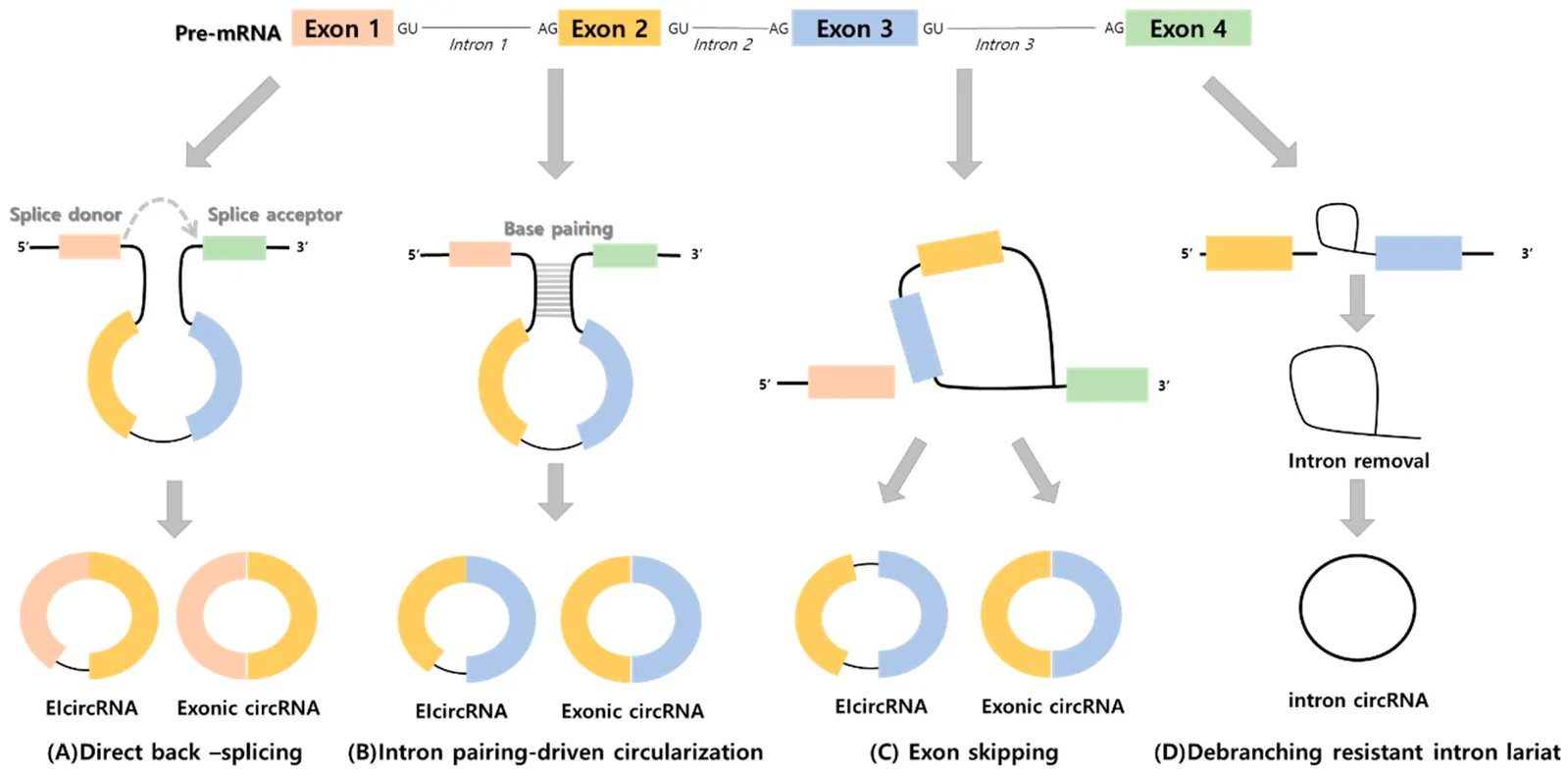
Biosynthetic mechanisms of circular RNAs. Circular RNAs are produced via several induction mechanisms: (**A**) direct back-splicing, (**B**) intron-pairing-driven circularization, (**C**) exon skipping, and (**D**) debranching-resistant intron lariat. EIcircRNA; Exon-intron circRNA.

The circRNAs are completely closed formed endogenous RNAs. Recent studies have shown that circRNAs are crucial modulatory factors of miRNAs, including MDTEs, through their biofunction as miRNA sponges to proficiently subtract miRNAs and proteins [24,28]. Advancements in high-throughput sequencing techniques have revealed that circRNAs are stable, abundant, and highly conserved across species [29]. Aberrantly expressed circRNAs in a tissue-specific manner have a distinct association with carcinogenesis via perturbation of cell proliferation, migration, and angiogenesis processes [30,31]. The circRNA contributes to the creation of a microenvironment by forming a complex network with miRNAs, their target genes, and transgenic genes that are regulated by related signaling cascades. Based on their biogenetic patterns, circRNAs can be separated into exonic circRNAs, intronic RNAs, and exon-intron circRNAs (EIcircRNA) [32,33]. These circRNAs are produced by diverse synthetic mechanisms: (1) direct back-splicing [34] (2) intron-pairing-driven circularization [35] (3) exon skipping [36] (4) branching-resistant intron lariat [37]. A general schematic illustration of circRNA biogenesis is shown in Figure 1. 

Although previous studies on the roles of the circRNA-MDTE axis in cancer are still limited, several research teams have conducted the expression profiles and advanced analysis of circRNA-miRNA regulatory networks in numerous cancer types. The aim of this review is to summarize the recent studies on circRNAs, including their biogenesis, function, and pathological relevance, through interactions with several miRNAs in cancer. Next, we discuss the biological importance of the regulatory axis composed of circRNAs, miRNAs, and target mRNA in tumorigenesis. Especially, we suggest a biological connection between transposable elements and circRNAs by introducing some MDTEs which have a close association with the onset and development of various cancer types.

## 2. Tumorigenic Regulation of Circular RNA–miRNA Axis in Five Major Mortality Cancers

**Figure 2:**
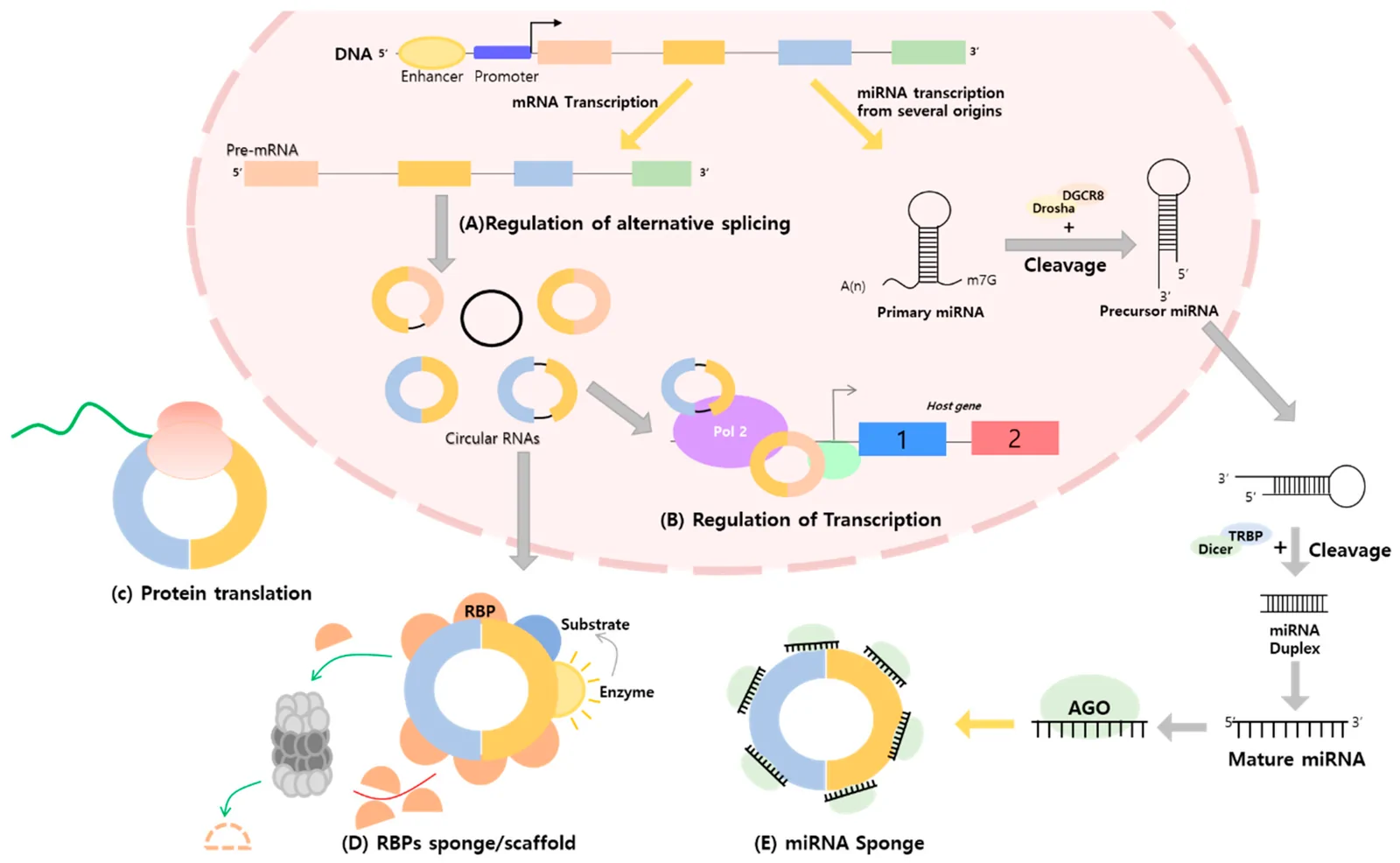
Regulatory processes that involve circular RNAs at the molecular level. Circular RNAs act as significant regulators by interacting with several components that control the vital biological processes, such as: (**A**) Regulation of alternative splicing, (**B**) Regulation of transcription, (**C**) Protein translation, (**D**) RBPs sponge/scaffold (**E**) miRNA sponge. pol 2, RNA polymerase 2; RBP, RNA-binding protein; AGO, argonaute protein.

Although only a few functions have been revealed thus far, many studies have identified that circRNAs are related to a wide range of cellular processes as major regulators of oncogenic factors (Figure 2). Some circRNAs localized in the nucleus could function as regulators of alternative splicing (Figure 2A) [38]. The generation of circRNAs produced by an exon-skipping mechanism directly affects alternative splicing of the originated gene and indirectly affects alternative splicing events of several genes by regulating the expression or activities of various splicing-related factors [39,40,41]. Furthermore, nucleic circRNAs have also been demonstrated to modulate gene expression at the transcriptional level by combining with U1 small nuclear ribonucleoprotein (U1 snRNP), which can promote the activity of the RNA polymerase II (Pol II) complex or recruit methyl-cytosine dioxygenase TET1 to the promoter region (Figure 2B) [42,43,44]. 

Owing to the deficiency of polyadenylated tails, 5′-3′ polarity and internal ribosome entry sites, circRNAs were generally defined as a kind of endogenous non-coding RNA molecules that could not be translated into proteins. However, some recent studies have also identified that they can be expressed into protein fragments via the rolling circle translation mechanism [45,46,47] (Figure 2C). The protein isoform formed by circRNAs would have unique parts of polypeptides for the isoform encoded by the specific sequences that are only of the circRNAs as well as common parts with the linearly encoded protein [48]. This suggests that the novel reading frames generated from circRNAs would expand the range of protein isoforms in cells [49]. Since the majority of the identified circRNAs are located in the cytoplasm, the most notable function of circRNAs is their activity as sponges for several cytoplasmic components [50,51]. Some cytoplasmic circRNAs can serve as sponges for RNA-binding proteins (RBPs) and then separate them from their targets or control the activity/stability of RBPs by forming RNA-protein complexes (RPCs). These RPCs could exert mutual effects with their linear RNA counterparts [52,53]. Indeed, other cytoplasmic circRNAs also act as protein scaffolds that enhance the reaction kinetics of certain enzymes and their substrates by inducing their colocalization (Figure 2D) [54,55].

In particular, multiple studies have proven that a number of circRNAs and mRNAs can compete for binding to the target miRNAs to build competing endogenous RNA (ceRNA) regulatory networks [56,57]. When miRNAs competitively bind to mRNAs, their translation is inhibited. In contrast, when circRNAs that possess miRNA recognition elements (MREs) bind to miRNAs, the activity of miRNAs is inhibited and the expression of mRNAs increases through interaction with miRNA-Ago2 complexes, called a “miRNA sponge mechanism” (Figure 2E) [58,59]. Furthermore, some circRNAs have a superior miRNA-binding ability compared to the other miRNA sponges, referred to as “super sponges” [60]. According to previous studies, the expressed level of miRNA is mostly regulated by direct binding of circRNA (miRNA sponge mechanism). In other words, the general function of miRNA, ‘suppression of mRNA expression’, is prevented by the circRNA regulatory mechanism (miRNA sponge), resulting in increased mRNA expression. Abnormal expression of miRNAs has also been reported in numerous malignancies, inducing alterations in biological pathways associated with cancer hallmarks [61]. Specific examples of circRNAs that control cancer-related miRNAs in the five major cancer-related mortalities over the last five years are summarized in Table 1. Based on the previous studies, circRNAs could be presented as promising cancer biomarkers for early diagnosis and efficient remedial targets for cancer therapy.

### 2.1. Lung Cancer

Lung cancer is one of the most prevalent cancer types worldwide, with the highest mortality rate (18% of the total cancer-related deaths) for both sexes, according to the global cancer report of 2020 [62]. In lung cancer progression, some circRNAs are known to form complex biological regulatory networks by simultaneously controlling multiple target genes. For example, oncogenic circ_103809 acts as a sponge for *miR-4302* and is involved in lung cancer cell proliferation and invasion in vitro and delayed tumor growth in vivo. Knockdown of *miR-4302* promotes the expression of *ZNF121* and consequently enhances ZNF121-dependent MYC expression [63]. Similarly, circ_HIPK3 has been known as a crucial oncogenic regulator in several cancer pathologies by suppressing two miRNAs, *miR-124* and *miR-149*. The downregulation of *miR-124* by circ_HIPK3 can promote cancer cell survival and proliferation by inducing the overexpression of its target genes, such as *SphK1, STAT3,* and *CDK4* [64]. Moreover, circ_HIPK3 can control the proliferation, migration, invasion, and apoptosis of lung cancer cells by binding *miR-149*-mediated FOXM1 expression [65].

In addition to carcinogenesis, some circRNAs are closely associated with therapeutic plans and poor prognosis of lung cancer. One study showed that patients overexpressing circ_PVT1 exhibited aggressive clinicopathological characteristics and poor prognosis due to circ_PVT1-mediated direct suppression of *miR-497* and indirect overexpression of *Bcl-2* [66]. Another study also identified that circ_ZNF208 was significantly upregulated in a radioresistant non-small cell lung cancer (NSCLC) cell line (A549-R11) compared to the normal control cell line (A549). Overexpression of circ_ZNF208 can interact with *miR-7-5p* and increase the expression of *SNCA,* which enhances the resistance of NSCLC cells to low linear energy transfer (LET) X-rays, which is not observed in NSCLC cells exposed to high-LET carbon ions [67]. Controversially, the expression of circ_cESRP1 was decreased in chemoresistant small cell lung cancer (SCLC) cell lines. The level of circ_cESRP1 has a positive relationship with drug sensitivity by directly binding to *miR-93-5p* and enhancing the downstream targets Smad7/p21(*CDKN1A*). The axis of circ_cESRP1-*miR-93-5p*-*CDKN1A* formed a negative feedback loop that controls the transforming growth factor-β (TGF-β) pathway. Consequently, pathogenic regulations mediate epithelial-mesenchymal transition and alter tumor responsiveness to chemotherapy in SCLC [68]. These circRNAs could be potential biomarkers and therapeutic targets for lung cancer treatment.

### 2.2. Colon Cancer

Colon cancer is a representative aggressive cancer type and the third leading cause of cancer-associated deaths worldwide in both sexes [62]. For an optimistic prognosis and cure of all types of cancers including colon cancer, studies identifying critical candidates as diagnostic biomarkers with high specificity and sensitivity are required for early detection. For example, upregulated circ_0001946 in colon cancer was negatively correlated with tumor size, histologic grade, lymphatic metastasis, and TMN stage which is an internationally recognized standard for classifying the extent of spread of cancer. Patients with high circ_0001946 expression are more likely to have a poor prognosis owing to the activation of the *miR-135a-5p/* epithelial mesenchymal transition (EMT) axis [47]. Additionally, in accordance with a study that performed several bioinformatic analyses, the decreased circ_0140388 (circ_HUWE1) could get significantly involved in lymphovascular invasion (*p* = 0.036), lymph node metastasis (*p* = 0.017), distant metastasis (*p* = 0.024), and TNM stage (*p* = 0.009), which is an internationally recognized standard for classifying the extent of the spread of a cancer. As a sponge for miR-486, circ_HUWE1 could serve as a potential therapeutic target and diagnostic biomarker for colon cancer [69]. The scientific value of circRNAs as potential biomarkers of colorectal cancer has been demonstrated in numerous studies [70,71,72,73]. 

Some circRNA studies have revealed that different circRNAs can promote cancer development by controlling the same miRNA. As a representative example, *miR-106b* acts as a tumorigenic factor in various oncogenic pathways that are commonly inhibited by circ_000984 and circ_0055625. The level of circ_000984 encoded by the *CDK6* gene are remarkably enhanced in colorectal cancer tissues and cell lines. As a ceRNA that competitively interacts with *miR106b*, circ_000984 could efficiently increase the expression of *CDK6*, thereby promoting a series of malignant phenotypes, such as proliferation, migration, and invasion [74]. Similarly, circ_0055625, one of the highly expressed ceRNAs in colon cancer tissues, is associated with the pathological TNM stage, metastasis by forming canonical regulatory axis with miR-106-5p and *ITGB8* [71]. In another example, the *miR-199* family is controlled by circ_NSD2 and circ_UBAP in colon cancer pathogenesis. According to the results of transcriptome sequencing, circ_NSD2 has been identified as a liver metastasis-associated circRNA in colon cancer that promotes migration and metastasis in vitro and in vivo. The circ-NSD2 has oncogenic characteristics by inhibiting tumor-suppressive miR-199b, thereby increasing the expression of *DDR1* and *JAG1*, which synergistically facilitates cell–matrix interaction, migration, and metastasis of colorectal cancer cell lines [75]. Additionally, repressed *miR-199a*, mediated by the overexpression of circ_UBAP2, could induce cell proliferation, migration, and invasion by upregulating *VEGFA* in colon cancer [76]. 

### 2.3. Liver Cancer

Liver cancer, the most common malignant tumor, ranked third in mortality rates in global cancer statistics for 2020 [62]. An increasing number of studies have verified that the aberrant expression of oncogenic circRNAs can affect liver cancer pathologies by binding to multiple target genes. In hepatocellular carcinoma (HCC), a subtype of liver cancer, circ_MET, which is derived from the chromosome 7q21-7q31 region, is an oncogenic marker for the initiation and development of HCC. The tumorigenic axis, *miR-30-5p*/Snail/ *DPP4*/*CXCL10* axis, is positively involved in the survival and recurrence of HCC by increasing EMT and the immunosuppressive tumor microenvironment [77]. Through GSE dataset analysis and in vitro experiments, another controlling network with circ_0001955/ *miR-516a-5p*/ *TRAF6* and *MAPK11* axis, it has also been reported that circ_0001955 can facilitate HCC tumor growth by sponging *miR-516a-5p* to boost *TRAF6* and *MAPK11* expression [78]. Similarly, another study has identified that upregulated circ_Cdr1as can enhance HCC cell proliferation and invasion, by forming an axis with downregulated *miR-7* and upregulating its target genes (*CCNE1* and *PIK3CD*) [47]. 

Based on previous liver cancer research, some tumor-suppressive circRNAs have been shown to have anti-cancer effects by mediating liver tumor growth. One study showed that the expression of circ_5692 at a significantly low level can ameliorate aggressive tumor growth via interaction with *miR-328-5p*, which was predicted to target the *DAB2IP* gene [79]. Another similar study identified that the lower expression level of circ_C3P1 can induce advanced TNM stage, tumor size, and invasion in HCC. The biological interaction between circ_C3P1 and *miR-464* enhances the expression of *PCK1*, resulting in tumor-suppressive regulation [80]. Downregulation of circ_HIAT1, also studied in clear cell renal cell carcinoma, promoted poor overall survival rates and cancer cell growth by sponging miR-3171 to increase the expression of *PTEN* [81]. 

### 2.4. Gastric Cancer

Gastric cancer (GC), which has the fourth highest cancer-related mortality rate for both sexes, is a malignant cancer derived from the gastric mucosal epithelium [62,82]. Recent studies have shown that circRNAs can be used as reliable biomarkers and therapeutic targets for gastric cancer. According to expression profiling by circRNA microarray, aberrantly expressed circ_PVRL3 is involved in the onset of GC, a higher TNM stage, and lower survival rates compared to the control. circ_PVRL3 was able to sponge nine miRNAs (*miR-203*, *miR-1272*, *miR-1283*, *miR-31*, *miR-638*, *miR-496*, *miR-485-3p*, *miR-766*, and *miR-876-3p*), which were predicted using binding prediction and annotation analysis [83].

Circular RNAs are divided into two types: oncogenic and tumor-suppressive circRNAs, depending on the type of effect on cancer development via regulation of specific target miRNAs and multiple target genes. The oncogenic circRNA circ_NHSL1 was positively related to clinicopathological traits and poor prognosis in patients with GC. To relieve the suppressive effect of *miR-1306-3p* on its target *SIX1* gene, circ_NHSL1 functions as an miRNA sponge and promoted SIX1 dependent-vimentin expression linked with cell mobility and invasion [84]. In contrast, as tumor-suppressive CircRNA and downregulated circ_CCDC9 in both GC tissues and cell lines were positively associated with aggravated cancer markers, such as tumor size, lymph node invasion, progressive clinical stage, and overall survival of GC patients. In the circ_CCDC9/ miR-6792-3p/CAV1 regulatory axis, GC progression is suppressed when circ_ CCDC9 and CAV1 are upregulated and *miR-6792-3p* is downregulated [85]. Considering the results of previous studies, various pathological changes are induced by an imbalance in the expression of oncogenic circRNAs and tumor-suppressive circRNAs in cancer-causing environments.

### 2.5. Breast Cancer 

Breast cancer is the most frequently diagnosed cancer type in women worldwide and is developed from the glandular epithelium of the breast [62,86]. Circular RNAs are emerging as sensitive and non-invasive biomarker candidates for the diagnosis and treatment of breast cancer. For example, circ_0008039 mediates a wide range of cancer development pathways by controlling numerous miRNA targets. Overexpression of circ_0008039 expedites proliferation, migration, and invasion of breast cancer cells by competitively binding to *miR-515-5p* and CBX4 expression [87]. Additionally, other research has shown that circ_0008039 could also promote the proliferation, cell-cycle progression and migration by serving as a ceRNA of *miR-432-5p* and elevating E2F3 expression [88].

Furthermore, some studies have revealed that several circRNAs play an oncogenic role by increasing tumorigenic-regulating glycolysis in breast cancer, which is closely correlated with the Warburg effect. For instance, one breast cancer study identified that overexpressed circ_0001982 activates glycolysis and tumor growth by binding to *miR-1287-5p*, which controls the expression of *MUC19* gene under hypoxia [47]. In another study, circRNA microarray sequencing and cell line experiments suggested that circ_RNF20 is upregulated in breast cancer which is related to poor clinical outcomes by inducing proliferation and the Warburg effect (aerobic glycolysis). Functionally, the higher expression of circ_RNF20 increased the level of *miR-487a* and decreased the level of its target gene, *HIF-1α*, that binds to the promoter of hexokinase II (HK2) [89].

Moreover, specific circRNAs are closely associated with metastasis in certain tissues. circ_BCBM1, a suppressor of miR-125a, plays an oncogenic role in breast cancer by shortening brain metastasis-free survival in breast cancer patients. According to its overexpression in breast cancer, the function of *miR-125a* was repressed, which hinders the expression of BRD4, inducing indirect suppression of *MMP9* via the Sonic hedgehog (SHH) signaling pathway [90].

**Table 1:** List of differentially expressed circular RNAs and its target miRNAs in five major mortality cancers in the previous 5 years.

Cancer Type	CircRNA Symbol /CircBase ID	Oncogenic /Tumor-Suppressive	MicroRNA	Target Gene	Cancer Related Biological/Regulatory Process	Ref.
Lung cancer	Circ_0000284	oncogenic	miR-377-3p	*PD-L1*	-	[47]
	Circ_0000376	oncogenic	miR-1182	*NOVA2*	glycolysis, viability, migration, and invasion	[91]
	Circ_0000677	oncogenic	miR-106b-5p	*CCND1*	proliferation	[92]
	Circ_0020123	oncogenic	miR-144	*ZEB1, EZH2*	growth and metastasis	[93]
	Circ_0087862	oncogenic	miR-1253	*RAB3D*	tumor growth	[94]
	Circ_BANP	oncogenic	miR-503	*LARP1*	cancer growth, migration and invasion	[95]
	Circ_MAN2B2	oncogenic	miR-1275	*FOXK1*	cell proliferation and invasion	[96]
	Circ_PIP5K1A	oncogenic	miR-600	*HIF* *-1* *α*	proliferation and metastasis	[97]
	Circ_CDR1	oncogenic	miR-641	*HOXA9*	cell stemness	[98]
	Circ_103809	oncogenic	miR-4302	*MYC*	cell proliferation and invasion	[63]
				*ZNF121*		
	Circ_HIPK3	oncogenic	miR-124	*SphK1, STAT3, CDK4*	cell survival, proliferation, cell death and apoptosis	[64]
			miR-149	*FOXM1*	cell proliferation and apoptosis	[65]
	Circ_PVT1	oncogenic	miR-497	*Bcl-2*	aggressive clinicopathological characteristics and poor prognosis	[66]
	Circ_ZNF208	oncogenic	miR-7-5p	*SNCA*	radio-sensitivity of patients to X-rays	[67]
	Circ_0046264	Tumor-suppressive	miR-1245	*BRCA2*	apoptosis, proliferation and invasion	[99]
	Circ_100395	Tumor-suppressive	miR-1228	*TCF21*	proliferation, migration and invasion	[100]
	Circ_cESRP1	Tumor-suppressive	miR-93-5p	*CDKN1A*	sensitivity to chemotherapy	[68]
	Circ_FOXO3	Tumor-suppressive	miR-155	*FOXO3*	cell proliferation, migration and invasion	[101]
Colon cancer	Circ_ERBIN	oncogenic	miR-125a-5p, miR-138-5p	*4EBP-1*	growth and metastasis	[102]
	Circ_0000467	oncogenic	miR-4766-5p	*KLF12*	proliferation, metastasis, and angiogenesis	[103]
	Circ_0001313	oncogenic	miR-510-5p	*AKT2*	proliferation and apoptosis	[70]
	Circ_0001946	oncogenic	miR-135a-5p	*-*	proliferation and metastasis	[104]
	Circ_0004277	oncogenic	miR-512-5p	*PTMA*	cell apoptosis and cell proliferation	[105]
	Circ_0005963	oncogenic	miR-122	*PKM2*	chemoresistance	[106]
	Circ_0007843	oncogenic	mIR-518c-5p	*MMP2*	migration and invasion	[107]
	Circ_000984	oncogenic	miR-106b	*CDK6*	cancer growth and metastasis	[74]
	Circ_0053277	oncogenic	miR-2467-3p	*MMP14*	cell proliferation, migration, and EMT	[108]
	Circ_0055625	oncogenic	miR-106b-5p	*ITGB8*	cell growth	[71]
	Circ_0060745	oncogenic	miR-4736	*CSE1L*	proliferation and metastasis	[109]
	Circ_CTNNA1	oncogenic	miR-149-5p	*FOXM1*	proliferation and invasion	[110]
	Circ_HIPK3	oncogenic	miR-7	*c-Myb*	cancer growth and metastasis	[111]
	Circ_HUWE1	oncogenic	miR-486	*-*	cell proliferation, migration and invasion	[69]
	Circ_NSD2	oncogenic	miR-199b-5p	*DDR1, JAG1*	migration and metastasis	[75]
	Circ_PRMT5	oncogenic	miR-377	*E2F3*	proliferation	[112]
	Circ_UBAP2	oncogenic	miR-199a	*VEGFA*	cell proliferation, migration, and invasion	[76]
	Circ_ACAP2	Tumor-suppressive	miR-21-5p	*Tiam1*	proliferation, migration, and invasion	[113]
Liver cancer	Circ_0003141	oncogenic	miR-1827	*UBAP2*	proliferation and invasion	[114]
	Circ_0061395	oncogenic	miR-877-5p	*PIK3R3*	proliferation, invasion, and migration	[115]
	Circ_FBLIM1	oncogenic	miR-346	*FBLIM1*	cell proliferation, apoptosis and invasion	[116]
	Circ_MAST1	oncogenic	miR-1299	*CTNND1*	cell proliferation and migration	[117]
	Circ_MET	oncogenic	miR-30-5p	*Snail, DPP4, CXCL10*	epithelial to mesenchymal transition	[77]
	Circ_ZNF566	oncogenic	miR-4738-3p	*TDO2*	cell migration, invasion, and proliferation	[118]
	circ_0000673	oncogenic	miR-767-3p	*SET*	cell proliferation and invasion	[119]
	Circ_0001955	oncogenic	miR-516a-5p	*TRAF6, MAPK11*	tumor growth	[78]
	Circ_0016788	oncogenic	miR-486	*CDK4*	proliferation, invasion and apoptosis	[120]
	Circ_0067934	oncogenic	miR-1324	*FZD5*	proliferation, migration and invasion	[121]
	Circ_103809	oncogenic	miR-377-3p	*FGFR1*	proliferation, cycle progression and migration	[122]
	Circ_104348	oncogenic	miR-187-3p	*RTKN2*	proliferation, migration, invasion and apoptosis	[123]
	Circ_GFRA1	oncogenic	miR-498	*NAP1L3*	growth and invasion	[124]
	Circ_HIPK3	oncogenic	miR-124	*AQP3*	cell proliferation and migration	[125]
	Circ_MAT2B	oncogenic	miR-338-3p	*PKM2*	glycolysis	[126]
	Circ_5692	Tumor-suppressive	miR-328-5p	*DAB2IP*	tumor growth	[79]
	Circ_C3P1	Tumor-suppressive	miR-4641	*PCK1*	tumor growth and metastasis	[80]
	Circ_HIAT1	Tumor-suppressive	miR-3171	*PTEN*	cell growth	[81]
Gastric cancer	Circ_0006282	oncogenic	miR-155	*FBXO22*	cell growth, proliferation and metastasis	[127]
	Circ_0008035	oncogenic	miR-375	*YBX1*	proliferation and invasion	[128]
	Circ_AKT3	oncogenic	miR-198	*PIK3R1*	DNA damage repair and apoptosis	[129]
	Circ_CACTIN	oncogenic	miR-331-3p	*TGFBR1*	tumor growth and EMT	[130]
	Circ_DLG1	oncogenic	miR-141-3p	*CXCL12*	proliferation, migration, invasion and immune evasion	[131]
	Circ_NHSL1	oncogenic	miR-1306-3p	*SIX1, vimentin*	cell mobility, invasion and metastasis.	[84]
	Circ_NRIP1	oncogenic	miR-149-5p	*AKT1*	proliferation, migration and invasion	[132]
	Circ_PDSS1	oncogenic	miR-186-5p	*NEK2*	cell cycle and apoptosis	[133]
	Circ_PVRL3	oncogenic	miR-203, miR-1272, miR-1283, miR-31, miR-638, miR-496, miR-485-3p, miR-766, and miR-876-3p	*-*	proliferation and migration	[83]
	Circ_0000039	oncogenic	miR-1292-5p	*DEK*	proliferation, migration and invasion	[134]
	Circ_RanGAP1	oncogenic	miR-877–3p	*VEGFA*	invasion and metastasis	[135]
	ciRS-7	oncogenic	miR-7	*PTEN, PI3K*	poor survival rate	[136]
	Circ_0026344	Tumor-suppressive	miR-590-5p	*PDCD4*	cell proliferation, migration and invasion	[137]
	Circ_CCDC9	Tumor-suppressive	miR-6792-3p	*CAV1*	tumor size, lymph node invasion, advanced clinical stage and survival rate	[85]
	Circ_LARP4	Tumor-suppressive	miR-424-5p	*LATS1*	cell proliferation and invasion	[138]
	Circ_MCTP2	Tumor-suppressive	miR-99a-5p	*MTMR3*	proliferation while promoting apoptosis of CDDP-resistant GC cells	[139]
Breast cancer	Circ_0001982	oncogenic	miR-1287-5p	*MUC19*	glycolysis, proliferation, migration, and invasion	[140]
	Circ_0001429	oncogenic	miR-205	*KDM4A*	metastasis	[141]
	Circ_0005230	oncogenic	miR-618	*CBX8*	prognostic predictor	[142]
	Circ_0008039	oncogenic	miR-515-5p	*CBX4*	proliferation, migration and invasion	[87]
	Circ_0008039	oncogenic	miR-432-5p	*E2F3*	proliferation, cell-cycle progression and migration	[88]
	Circ_0136666	oncogenic	miR-1299	*CDK6*	proliferation, migration and invasion	[143]
	Circ_100219	oncogenic	miR-485-3p	*NTRK3*	proliferation and migration	[144]
	Circ_ANKS1B	oncogenic	miR-148a-3p, miR-152-3p	*USF1*	metastasis	[145]
	Circ_BCBM1	oncogenic	miR-125a	*BRD4*	breast cancer brain metastasis	[90]
	Circ_CER	oncogenic	miR-136	*MMP13*	cell proliferation and migration	[146]
	Circ_FOXK2	oncogenic	miR-370	*IGF2BP3*	migration and invasion	[147]
	Circ_MYO9B	oncogenic	miR-4316	*FOXP4*	cell proliferation and invasion	[148]
	Circ_PLK1	oncogenic	miR-4500	*IGF1*	cell proliferation, migration and invasion	[149]
	Circ_RHOT1	oncogenic	miR-106a-5p	*STAT3*	malignant progression and ferroptosis	[150]
	Circ_RNF20	oncogenic	miR-487a	*HIF-1* *α*	proliferation, Warburg effect	[89]
	Circ_RPPH1	oncogenic	miR-556-5p	*YAP1*	proliferation, migration, invasion, and angiogenesis	[151]
	Circ_HIPK3	oncogenic	miR-193a	*HMGB1, PI3K, AKT*	cell proliferation and invasion	[152]
	Circ_0000442	Tumor-suppressive	miR-148b-3p	*PTEN*	tumor growth	[153]
	Circ_CCDC85A	Tumor-suppressive	miR-550a-5p	*MOB1A*	proliferation, migration and invasion	[154]

## 3. MDTEs Regulated by Oncogenic CircRNAs and Tumor-Suppressive circ RNAs in Various Cancers

Recent studies have also identified that many of these miRNAs are derived from transposable elements (known as MDTEs) and function as crucial tumorigenic factors in cancer development. Numerous MDTEs induce carcinogenesis by directly or indirectly interacting with cancer-related genes. One research study has shown that the upregulated *miR-1269* derived from LTR elements could constrain the expression of *RASSF9,* which is closely concerned with the AKT and Bax/Bcl-2 signaling pathway, resulting in GC progression [155]. In contrast, other studies have identified that *miR-1273g* originating from the short interspersed nuclear element (SINE) acts as a tumor-suppressor miR in colon cancer by promoting MAGEA3/6 and inhibiting *AMPKα1* [156]. Furthermore, MDTEs are known to contribute to tumorigenesis through biological associations with non-coding RNAs such as circRNAs. The list of MDTEs regulated by oncogenic or tumor-suppressive circRNAs is summarized in Table 2.

### 3.1. MDTEs That Play a Common Role in Various Types of Cancer

According to previous reports, some miRNAs originating from transposons can act as communal controllers in numerous cancers. For example, miR-326 is derived from DNA transposon, a type of transposable element. Downregulated *miR-326* by oncogenic circRNAs had a close association with tumerigenic progresses in lung cancer, liver cancer and breast cancer. In lung cancer, increased circ_0003998 and circ_POLA2 regulate cell proliferation and invasion, resulting in a poor survival rate in lung cancer patients relying on miR-326, which suppresses its target genes, *Notch1* and *GNB1*. These circRNAs may serve as novel therapeutic targets for patients with lung cancer. [157,158]. Liver cancer studies have also identified that relatively overexpressed circ_0000517 is involved in cancer cell viability, colony formation, migration, invasion, and glycolysis via modulation of *miR-326* and the target gene *IGF1R* [159]. Furthermore, the expressed level of *miR-326* is inhibited by circ_0061825, also known as circ_TFF1, resulting in the overexpression of the *TFF1* gene in breast cancer. These regulations can induce breast cancer cell proliferation, migration, invasion, and EMT in vitro and regulate tumor growth in vivo [160]. In another representative case, *miR-637*, derived from long interspersed nuclear element (LINE), regulates cell cancer growth and metastasis by directly binding to several oncogenic circRNAs. As a tumorigenic ceRNA, circ-0000284 stimulates carcinogenic processes related to cholangiocarcinoma progression by directly binding *miR-637*. Moreover, circ-0000284 transferred to adjacent normal cells is also adjusted for general biological functions [161]. In addition, other studies have shown that the significantly increased expression of circ_HIPK3 may play an oncogenic role in GC. In accordance with experimental and bioinformatic analyses, circ_HIPK3 could form a controlling axis with miR-637 and its direct target gene *AKT1* [162]. A related study on circ_HIPK3 also demonstrated that cytoplasmic circ_HIPK3 could bind to its downstream target, *HDAC4* in osteosarcoma. As a result of this research, the circHIPK3/*miR-637*/HDAC4 axis could control the proliferation, migration, and invasion of osteosarcoma cells [163]. Conversely, *miR-421* created from LINE (L2) has been fine-tuned by numerous tumor-suppressive circRNAs. In liver cancer, circ_SETD3 (hsa_circRNA_0000567, also known as hsa_circRNA_101436) acts as a *miR-421* sponge for tumor suppression. Dysregulated expression of circ_SETD3 and *miR-421* was predicted to be a risk factor for poor prognosis and a larger tumor size. In a related study, circ_SETD3 induced G1/S arrest via coupling with *miR-421* and *MAPK14* in a liver cancer cell line [164]. Another study showed that *miR-421* is positively associated with triple-negative breast cancer, an aggressive subtype of breast cancer, through its connection with circ_AHNAK1. Downregulated circ_AHNAK1 has been shown to restrict its tumor-suppressive function in breast cancer, which modulates the expression of oncogenic miR-421 and its target gene *RASA1* [165].

### 3.2. MDTEs That Play Diverse Roles Depending on Regulation of Specific CircRNAs in Several Cancers

Some MDTEs can control tumorigenic or tumor-suppressive biological processes in various cancers, depending on their direct interactions with specific circRNAs. In regulatory networks composed of circRNA, MDTE, and its target genes, MDTEs act as vital modulators in cancer progression. Reportedly, *miR-224* generated from DNA transposon sequences regulates oncogenic or tumor-suppressive circRNAs in gastric and bladder cancers. In bladder cancer, *miR-224* is controlled by combining with the tumor-suppressive circRNA, circ_ITCH, which suppresses the expression of *miR-17* and *miR-224* and upregulates its target genes *p21* and *PTEN*. Downregulated circ_ITCH was associated with a poor survival rate by inducing proliferation, migration, invasion, and metastasis of bladder cancer cells [166]. Similarly, *miR-224* was positively upregulated and suppressed its target genes containing *cyclin D1, CDK6, MMP-2* and *MMP-9* by the low level of tumor-suppressive circ_0000096 in GC [167]. In contrast, overexpression of oncogenic circRNA circ_ LDLRAD3 controlled cell growth, migration, invasion, and apoptosis in GC by directly suppressing the expression of *miR-224* and increasing the expression of the downstream target gene *NRP2* [168]. Other studies have also revealed that miR-330, originating from SINE sequences, has a direct connection with tumor-suppressive circ_0078767 and oncogenic circ-ZKSCAN1 in NSCLC. In a bioinformatic analysis, circ_0078767 and the target genes of *miR-330* and *RASSF1A* were downregulated, whereas *miR-330* was upregulated compared to adjacent normal tissue. These alterations can suppress NSCLC cell viability, cell cycle progression, and invasion, and promote cell apoptosis [169]. In contrast, overexpressed circ-ZKSCAN1 can function as an miRNA sponge of *miR-330-5p* to increase the expression of *FAM83A*, thereby promoting the inhibition of the MAPK signal transduction pathway. The feedback loop among oncogenic circRNAs, circ-ZKSCAN1, *miR-330-5p*, and *FAM83A* is closely related to malignant characteristics, such as poor prognosis, larger tumor size, and advanced clinical stage [170]. Moreover, recent studies have identified that the level of *miR-330* can be inhibited by the same circRNA, circ-FARSA, in different types of cancer. Upregulated circ-FARSA enhances the proliferation, migration, and invasion of colon cancer cells by sponging *miR-330-5p*. Its upregulation attenuates the inhibitory effects of *miR-330-5p* on cell proliferation and metastasis by increasing *LASP1* expression [171]. In bladder cancer, circ_FARSA can also act as an oncogenic circRNA contributing to cancer hallmarks, including cell proliferation, invasion, apoptosis, and migration. *miR-330-5p* is directly suppressed by circ_ FARSA and inhibits its activity as a regulator of target gene [172].

**Table 2:** List of miRNAs derived from transposable element (MDTEs) regulated by oncogenic and tumor-suppressive circular RNAs and its dysregulated target genes in various cancer types.

Type of Circular RNA	Cancer Type	CircRNA Symbol /CircBase ID	MicroRNA	Subclass	Super Family	Target Gene	Cancer-Related Regulatory Process	Ref.
Oncogenic circular RNA	Bladder cancer	Circ_FARSA	miR-330-5p	SINE	MIR	*-*	cell proliferation, invasion, apoptosis and migration	[172]
	Breast cancer	Circ_0007255	miR-335-5p	SINE	MIR	*SIX2*	inhibition of oxygen consumption, colony formation, cell migration and invasion	[173]
		Circ_0061825 (circ-TFF1)	miR-326	DNA transposon	hAT-Tip100	*TFF1*	cell proliferation, migration, invasion and EMT	[160]
		Circ_ABCB10	miR-1271	LINE	L2	*-*	proliferation and apoptosis	[174]
	Cervical cancer	Circ_0067934	miR-545	LINE	L2	*EIF3C*	proliferation, colony formation, migration, invasion and EMT	[175]
		Circ_0141539 (Circ_8924)	miR-518d-5p	LINE	RTE-BovB	*CBX8*	cell proliferation, migration and invasion	[176]
	Cholangiocarcinoma	Circ_0000284	miR-637	LINE	L1	*-*	migration, invasion and proliferation	[161]
	Chronic lymphocytic leukemia	Circ_CBFB	miR-607	SINE	MIR	*FZD3*	proliferation and apoptosis	[177]
	Colon cancer	Circ_PIP5K1A	miR-1273a	SINE	Alu	*AP-1, IRF-4, CDX-2, Zic-1*	cell viability, cell invasion and migration	[178]
		Circ_FARSA	miR-330-5p	SINE	MIR	*LASP1*	cell growth	[171]
		Circ_ALG1	miR-342-5p	SINE	tRNA-RTE	*PGF*	metastasis	[179]
		Circ_102958	miR-585	LTR	ERVL-MaLR	*CDC25B*	growth, migration and invasion	[180]
	Glioma	Circ_0001982	miR-1205	SINE	MIR	*E2F1*	cell proliferation, migration, invasion and cell cycle progression	[181]
		Circ_0034642	miR-1205	SINE	MIR	*BATF3*	cell proliferation and invasion	[182]
	Liver cancer	Circ_0000517	miR-326	DNA transposon	hAT-Tip100	*IGF1R*	cell viability, colony formation, migration, invasion and glycolysis	[159]
		Circ_G004213	miR-513b-5p	DNA transposon	hAT-Tip100	*PRPF39*	cisplatin sensitivity and prognosis	[183]
		Circ_ 001306	miR-584-5p	DNA transposon	hAT-Blackjack	*CDK16*	cell proliferation and growth	[184]
		Circ_ 104075	miR-582-3p	LINE	CR1	*HNF4a*	YAP-dependent tumorigenesis	[185]
	Lung cancer	Circ_ FOXM1	miR-1304-5p	SINE	Alu	*PPDPF, MACC1*	proliferation and invasion	[186]
		Circ_ ZKSCAN1	miR-330-5p	SINE	MIR	*FAM83A*	tumor growth	[170]
		Circ_ 0004015	miR-1183	LINE	L2	*PDPK1*	proliferation, invasion, TKI drug resistance	[187]
		Circ_ 0014130	miR-493-5p	LINE	L2	*-*	-	[188]
		Circ_ POLA2	miR-326	DNA transposon	hAT-Tip100	*GNB1*	cell stemness and progression	[158]
		Circ_ 0003998	miR-326	DNA transposon	hAT-Tip100	*Notch1*	cell proliferation and invasion	[157]
	Osteosarcoma	Circ_ HIPK3	miR-637	LINE	L1	*HDAC4*	proliferation and migration and invasion	[163]
	Papillary thyroid cancer	Circ_ ZFR	miR-1261	DNA transposon	TcMar-Tigger	*C8orf4*	cell proliferation and invasion	[189]
	Stomach cancer	Circ-LDLRAD3	miR-224-5p	DNA transposon	DNA transposon	*-*	cell growth, migration invasion and apoptosis	[168]
		Circ_HIPK3	miR-637	LINE	L1	*AKT1*	cell growth and metastasis	[162]
Circ_0008287		miR-548c-3p	DNA transposon	TcMar-Mariner	*CLIC1*	immune escape of cancer cell	[190]
Tumor- Suppressive circular RNA	Bladder cancer	Circ_ITCH	miR-224	DNA transposon	DNA transposon	*p21, PTEN*	cell proliferation, migration, invasion and metastasis	[166]
		Circ_HIPK3	miR-558	LTR	ERVL-MaLR	*HPSE*	migration, invasion, and angiogenesis	[191]
	Breast cancer	Circ_AHNAK1	miR-421	LINE	L2	*RASA1*	proliferation and metastasis	[165]
	Colon cancer	Circ_SMARCA5	miR-552	LINE	L1	*-*	growth, migration and invasion	[192]
	Liver cancer	Circ_SETD3 (circ_0000567)	miR-421	LINE	L2	*MAPK14*	tumor cell growth	[164]
	Lung cancer	Circ_0078767	miR-330-5p	SINE	MIR	*RASSF1A*	cancer cell viability, cell cycle progression and invasion	[169]
		Circ_0007059	miR-378	SINE	MIR	*p53, CyclinD1, Bax, Cleaved-Caspase-3, E-cadherin, Vimentin, Twist, Zeb1*	proliferation and EMT	[193]
	Osteosarcoma	Circ_0002052	miR-1205	SINE	MIR	*APC2*	cell proliferation, migration, invasion and apoptosis	[194]
	Stomach cancer	Circ_FAT1(e2)	miR-548g	DNA transposon	TcMar-Mariner	*YBX1*	cell proliferation, migration and invasion	[195]
		Circ_0000096	miR-224	DNA transposon	DNA transposon	*cyclin D1, CDK6, MMP-2, MMP-9*	cell growth and migration	[167]

## 4. Conclusions

**Figure 3:**
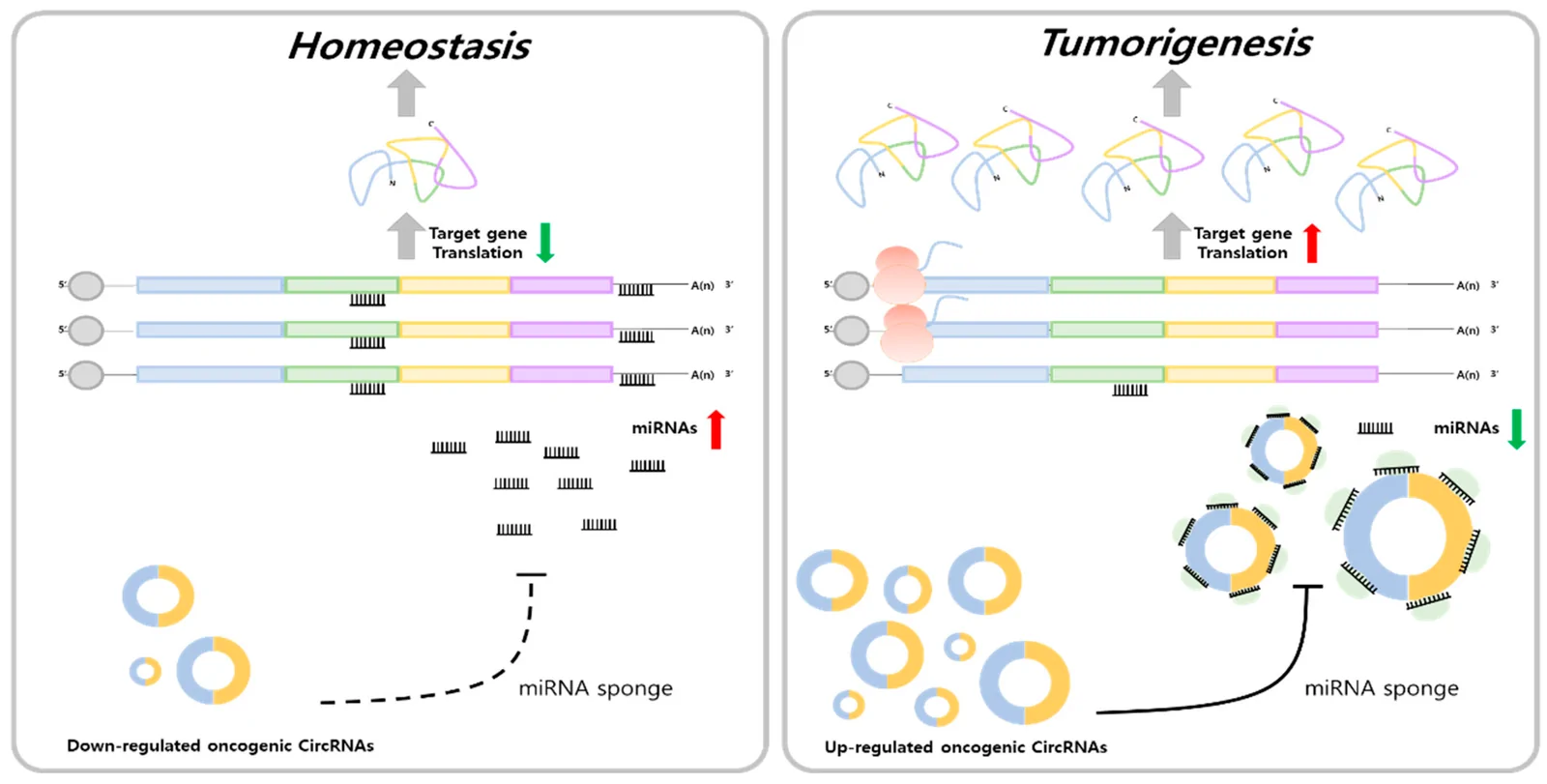
Impact of correlation between oncogenic circular RNA and miRNA in cancer.

Various cancer studies have revealed that atypical expression levels of miRNAs are closely associated with the development and progression of cancer. Among them, some miRNAs originate from transposble elements that can change their location in the genome and provide biological diversity, including the occurrence of numerous diseases. circRNAs are crucial suppressors of miRNAs, including MDTEs via miRNA sponge mechanisms that combine complementary with activated miRNAs. Under normal cellular conditions, the expression of oncogenic circRNAs is relatively low. Appropriately activated miRNAs suppress the translation of oncogenic target genes to sustain biological homeostasis. Conversely, the upregulated oncogenic circRNAs in the cancer environment can result in the inhibition of miRNA activity by inducing a miRNA-suppressive mechanism. The tumorigenic imbalance of the circRNA-miRNA axis abnormally increases the expression of oncogenic target genes, leading to several cancer pathologies (Figure 3). That is, the negative regulatory axis can adjust the expression of oncogenes in several tumorigenic signaling pathways as a major controlling factor. Despite their pathological importance, only a few studies have investigated the close regulatory relationship between circRNAs and miRNAs, especially MDTEs. The complete molecular mechanisms underlying the interplay between circRNAs and MDTEs in cancer need to be elucidated. In-depth studies of the circRNA-MDTE axis in tumorigenic status are needed to provide innovative perspectives of new actionable metabolic pathways for cancer treatment and a fundamental understanding of the complexity of central regulatory elements that form the oncogenic axis. 

## Data Availability

Not applicable.

## References

[1] Dong Y., Xu S., Liu J., Ponnusamy M., Zhao Y., Zhang Y., Wang Q., Li P., Wang K. (2018). Non-coding RNA-linked epigenetic regulation in cardiac hypertrophy. Int. J. Biol. Sci..

[2] Esteller M. (2011). Non-coding RNAs in human disease. Nat. Rev. Genet..

[3] Lekka E., Hall J. (2018). Noncoding RNA s in disease. FEBS Lett..

[4] Li M., Duan L., Li Y., Liu B. (2019). Long noncoding RNA/circular noncoding RNA–miRNA–mRNA axes in cardiovascular diseases. Life Sci..

[5] Su Q., Lv X. (2020). Revealing new landscape of cardiovascular disease through circular RNA-miRNA-mRNA axis. Genomics.

[6] Guo L., Jia L., Luo L., Xu X., Xiang Y., Ren Y., Ren D., Shen L., Liang T. (2022). Critical roles of circular RNA in tumor metastasis via acting as a sponge of miRNA/isomiR. Int. J. Mol. Sci..

[7] Cheng D., Wang J., Dong Z., Li X. (2021). Cancer-related circular RNA: Diverse biological functions. Cancer Cell Int..

[8] Marinescu M.-C., Lazar A.-L., Marta M.M., Cozma A., Catana C.-S. (2022). Non-Coding RNAs: Prevention, Diagnosis, and Treatment in Myocardial Ischemia–Reperfusion Injury. Int. J. Mol. Sci..

[9] Paul S., Ruiz-Manriquez L.M., Ledesma-Pacheco S.J., Benavides-Aguilar J.A., Torres-Copado A., Morales-Rodríguez J.I., De Donato M., Srivastava A. (2021). Roles of microRNAs in chronic pediatric diseases and their use as potential biomarkers: A review. Arch. Biochem. Biophys..

[10] Pandey M., Mukhopadhyay A., Sharawat S.K., Kumar S. (2021). Role of microRNAs in regulating cell proliferation, metastasis and chemoresistance and their applications as cancer biomarkers in small cell lung cancer. Biochim. Et Biophys. Acta (BBA)-Rev. Cancer.

[11] Ha M., Kim V.N. (2014). Regulation of microRNA biogenesis. Nat. Rev. Mol. Cell Biol..

[12] Campo-Paysaa F., Sémon M., Cameron R.A., Peterson K.J., Schubert M. (2011). microRNA complements in deuterostomes: Origin and evolution of microRNAs. Evol. Dev..

[13] Piriyapongsa J., Mariño-Ramírez L., Jordan I.K. (2007). Origin and evolution of human microRNAs from transposable elements. Genetics.

[14] Rani V., Sengar R.S. (2022). Biogenesis and mechanisms of microRNA-mediated gene regulation. Biotechnol. Bioeng..

[15] Hill M., Tran N. (2021). Global miRNA to miRNA interactions: Impacts for miR-21. Trends Cell Biol..

[16] Gong Y., Zhang X. (2021). RNAi-based antiviral immunity of shrimp. Dev. Comp. Immunol..

[17] Chen C., Zeng Z., Liu Z., Xia R. (2018). Small RNAs, emerging regulators critical for the development of horticultural traits. Hortic. Res..

[18] Alles J., Fehlmann T., Fischer U., Backes C., Galata V., Minet M., Hart M., Abu-Halima M., Grässer F.A., Lenhof H.-P. (2019). An estimate of the total number of true human miRNAs. Nucleic Acids Res..

[19] Fernández-Hernando C., Suárez Y. (2018). MicroRNAs in endothelial cell homeostasis and vascular disease. Curr. Opin. Hematol..

[20] Zhang Y., Li S., Jin P., Shang T., Sun R., Lu L., Guo K., Liu J., Tong Y., Wang J. (2022). Dual functions of microRNA-17 in maintaining cartilage homeostasis and protection against osteoarthritis. Nat. Commun..

[21] Qi X., Zhang D.-H., Wu N., Xiao J.-H., Wang X., Ma W. (2015). ceRNA in cancer: Possible functions and clinical implications. J. Med. Genet..

[22] Panda A.C. (2018). Circular RNAs act as miRNA sponges. Circ. RNAs.

[23] Cai X., Lin L., Zhang Q., Wu W., Su A. (2020). Bioinformatics analysis of the circRNA–miRNA–mRNA network for non-small cell lung cancer. J. Int. Med. Res..

[24] Ali A., Han K., Liang P. (2021). Role of transposable elements in gene regulation in the human genome. Life.

[25] Campo S., Sánchez-Sanuy F., Camargo-Ramírez R., Gómez-Ariza J., Baldrich P., Campos-Soriano L., Soto-Suárez M., San Segundo B. (2021). A novel Transposable element-derived microRNA participates in plant immunity to rice blast disease. Plant Biotechnol. J..

[26] Lee H.-E., Huh J.-W., Kim H.-S. (2020). Bioinformatics analysis of evolution and human disease related transposable element-derived microRNAs. Life.

[27] Anwar S.L., Wulaningsih W., Lehmann U. (2017). Transposable elements in human cancer: Causes and consequences of deregulation. Int. J. Mol. Sci..

[28] Prats A.-C., David F., Diallo L.H., Roussel E., Tatin F., Garmy-Susini B., Lacazette E. (2020). Circular RNA, the key for translation. Int. J. Mol. Sci..

[29] Meng X., Li X., Zhang P., Wang J., Zhou Y., Chen M. (2017). Circular RNA: An emerging key player in RNA world. Brief. Bioinform..

[30] Li W., Liu J.Q., Chen M., Xu J., Zhu D. (2022). Circular RNA in cancer development and immune regulation. J. Cell. Mol. Med..

[31] Kristensen L., Hansen T., Venø M., Kjems J. (2018). Circular RNAs in cancer: Opportunities and challenges in the field. Oncogene.

[32] Li M., Ding W., Sun T., Tariq M.A., Xu T., Li P., Wang J. (2018). Biogenesis of circular RNA s and their roles in cardiovascular development and pathology. FEBS J..

[33] Panda A.C., De S., Grammatikakis I., Munk R., Yang X., Piao Y., Dudekula D.B., Abdelmohsen K., Gorospe M. (2017). High-purity circular RNA isolation method (RPAD) reveals vast collection of intronic circRNAs. Nucleic Acids Res..

[34] Eger N., Schoppe L., Schuster S., Laufs U., Boeckel J.-N. (2018). Circular RNA splicing. Circ. RNAs.

[35] Yao T., Chen Q., Fu L., Guo J. (2017). Circular RNAs: Biogenesis, properties, roles, and their relationships with liver diseases. Hepatol. Res..

[36] Kelly S., Greenman C., Cook P.R., Papantonis A. (2015). Exon skipping is correlated with exon circularization. J. Mol. Biol..

[37] Li J., Yang J., Zhou P., Le Y., Zhou C., Wang S., Xu D., Lin H.-K., Gong Z. (2015). Circular RNAs in cancer: Novel insights into origins, properties, functions and implications. Am. J. Cancer Res..

[38] Huang M.-S., Zhu T., Li L., Xie P., Li X., Zhou H.-H., Liu Z.-Q. (2018). LncRNAs and CircRNAs from the same gene: Masterpieces of RNA splicing. Cancer Lett..

[39] Zhao W., Li M., Wang S., Li Z., Li H., Li S. (2022). CircRNA SRRM4 affects glucose metabolism by regulating PKM alternative splicing via SRSF3 deubiquitination in epilepsy. Neuropathol. Appl. Neurobiol..

[40] Gao Y., Wang J., Zheng Y., Zhang J., Chen S., Zhao F. (2016). Comprehensive identification of internal structure and alternative splicing events in circular RNAs. Nat. Commun..

[41] Aufiero S., van den Hoogenhof M.M., Reckman Y.J., Beqqali A., van der Made I., Kluin J., Khan M.A., Pinto Y.M., Creemers E.E. (2018). Cardiac circRNAs arise mainly from constitutive exons rather than alternatively spliced exons. RNA.

[42] Chen L.-L. (2016). The biogenesis and emerging roles of circular RNAs. Nat. Rev. Mol. Cell Biol..

[43] Li Z., Huang C., Bao C., Chen L., Lin M., Wang X., Zhong G., Yu B., Hu W., Dai L. (2015). Exon-intron circular RNAs regulate transcription in the nucleus. Nat. Struct. Mol. Biol..

[44] Chen N., Zhao G., Yan X., Lv Z., Yin H., Zhang S., Song W., Li X., Li L., Du Z. (2018). A novel FLI1 exonic circular RNA promotes metastasis in breast cancer by coordinately regulating TET1 and DNMT1. Genome Biol..

[45] Wang X., Fang L. (2018). Advances in circular RNAs and their roles in breast Cancer. J. Exp. Clin. Cancer Res..

[46] Liu D., Mewalal R., Hu R., Tuskan G.A., Yang X. (2017). New technologies accelerate the exploration of non-coding RNAs in horticultural plants. Hortic. Res..

[47] Abe N., Matsumoto K., Nishihara M., Nakano Y., Shibata A., Maruyama H., Shuto S., Matsuda A., Yoshida M., Ito Y. (2015). Rolling circle translation of circular RNA in living human cells. Sci. Rep..

[48] Wen S.-y., Qadir J., Yang B.B. (2022). Circular RNA translation: Novel protein isoforms and clinical significance. Trends Mol. Med..

[49] Pamudurti N.R., Bartok O., Jens M., Ashwal-Fluss R., Stottmeister C., Ruhe L., Hanan M., Wyler E., Perez-Hernandez D., Ramberger E. (2017). Translation of circRNAs. Mol. Cell.

[50] Memczak S., Jens M., Elefsinioti A., Torti F., Krueger J., Rybak A., Maier L., Mackowiak S.D., Gregersen L.H., Munschauer M. (2013). Circular RNAs are a large class of animal RNAs with regulatory potency. Nature.

[51] Zhang Y., Zhang X.-O., Chen T., Xiang J.-F., Yin Q.-F., Xing Y.-H., Zhu S., Yang L., Chen L.-L. (2013). Circular intronic long noncoding RNAs. Mol. Cell.

[52] Salzman J., Gawad C., Wang P.L., Lacayo N., Brown P.O. (2012). Circular RNAs are the predominant transcript isoform from hundreds of human genes in diverse cell types. PLoS ONE.

[53] Zhu L.-P., He Y.-J., Hou J.-C., Chen X., Zhou S.-Y., Yang S.-J., Li J., Zhang H.-D., Hu J.-H., Zhong S.-L. (2017). The role of circRNAs in cancers. Biosci. Rep..

[54] Zeng Y., Du W.W., Wu Y., Yang Z., Awan F.M., Li X., Yang W., Zhang C., Yang Q., Yee A. (2017). A circular RNA binds to and activates AKT phosphorylation and nuclear localization reducing apoptosis and enhancing cardiac repair. Theranostics.

[55] Du W.W., Fang L., Yang W., Wu N., Awan F.M., Yang Z., Yang B.B. (2017). Induction of tumor apoptosis through a circular RNA enhancing Foxo3 activity. Cell Death Differ..

[56] Chen J., Gu J., Tang M., Liao Z., Tang R., Zhou L., Su M., Jiang J., Hu Y., Chen Y. (2022). Regulation of cancer progression by circRNA and functional proteins. J. Cell. Physiol..

[57] Liang Z.-Z., Guo C., Zou M.-M., Meng P., Zhang T.-T. (2020). circRNA-miRNA-mRNA regulatory network in human lung cancer: An update. Cancer Cell Int..

[58] Das A., Sinha T., Shyamal S., Panda A.C. (2021). Emerging role of circular RNA–protein interactions. Non-Coding RNA.

[59] Bezzi M., Guarnerio J., Pandolfi P.P. (2017). A circular twist on microRNA regulation. Cell Res..

[60] Zhang M., Xin Y. (2018). Circular RNAs: A new frontier for cancer diagnosis and therapy. J. Hematol. Oncol..

[61] He Y., Lin J., Ding Y., Liu G., Luo Y., Huang M., Xu C., Kim T.K., Etheridge A., Lin M. (2016). A systematic study on dysregulated micro RNA s in cervical cancer development. Int. J. Cancer.

[62] Sung H., Ferlay J., Siegel R.L., Laversanne M., Soerjomataram I., Jemal A., Bray F. (2021). Global cancer statistics 2020: GLOBOCAN estimates of incidence and mortality worldwide for 36 cancers in 185 countries. CA A Cancer J. Clin..

[63] Liu W., Ma W., Yuan Y., Zhang Y., Sun S. (2018). Circular RNA hsa_circRNA_103809 promotes lung cancer progression via facilitating ZNF121-dependent MYC expression by sequestering miR-4302. Biochem. Biophys. Res. Commun..

[64] Yu H., Chen Y., Jiang P. (2018). Circular RNA HIPK3 exerts oncogenic properties through suppression of miR-124 in lung cancer. Biochem. Biophys. Res. Commun..

[65] Lu H., Han X., Ren J., Ren K., Li Z., Sun Z. (2020). Circular RNA HIPK3 induces cell proliferation and inhibits apoptosis in non-small cell lung cancer through sponging miR-149. Cancer Biol. Ther..

[66] Qin S., Zhao Y., Lim G., Lin H., Zhang X., Zhang X. (2019). Circular RNA PVT1 acts as a competing endogenous RNA for miR-497 in promoting non-small cell lung cancer progression. Biomed. Pharmacother..

[67] Liu B., Li H., Liu X., Li F., Chen W., Kuang Y., Zhao X., Li L., Yu B., Jin X. (2021). CircZNF208 enhances the sensitivity to X-rays instead of carbon-ions through the miR-7-5p/SNCA signal axis in non-small-cell lung cancer cells. Cell. Signal..

[68] Huang W., Yang Y., Wu J., Niu Y., Yao Y., Zhang J., Huang X., Liang S., Chen R., Chen S. (2020). Circular RNA cESRP1 sensitises small cell lung cancer cells to chemotherapy by sponging miR-93-5p to inhibit TGF-β signalling. Cell Death Differ..

[69] Chen H.-Y., Li X.-N., Ye C.-X., Chen Z.-L., Wang Z.-J. (2020). Circular RNA circHUWE1 is upregulated and promotes cell proliferation, migration and invasion in colorectal cancer by sponging miR-486. OncoTargets Ther..

[70] Tu F.-L., Guo X.-Q., Wu H.-X., He Z.-Y., Wang F., Sun A.-J., Dai X.-D. (2020). Circ-0001313/miRNA-510-5p/AKT2 axis promotes the development and progression of colon cancer. Am. J. Transl. Res..

[71] Zhang J., Liu H., Zhao P., Zhou H., Mao T. (2019). Has_circ_0055625 from circRNA profile increases colon cancer cell growth by sponging miR-106b-5p. J. Cell. Biochem..

[72] Xu H., Wang C., Song H., Xu Y., Ji G. (2019). RNA-Seq profiling of circular RNAs in human colorectal Cancer liver metastasis and the potential biomarkers. Mol. Cancer.

[73] Ji W., Qiu C., Wang M., Mao N., Wu S., Dai Y. (2018). Hsa_circ_0001649: A circular RNA and potential novel biomarker for colorectal cancer. Biochem. Biophys. Res. Commun..

[74] Xu X.-W., Zheng B.-A., Hu Z.-M., Qian Z.-Y., Huang C.-J., Liu X.-Q., Wu W.-D. (2017). Circular RNA hsa_circ_000984 promotes colon cancer growth and metastasis by sponging miR-106b. Oncotarget.

[75] Chen L.Y., Zhi Z., Wang L., Zhao Y.Y., Deng M., Liu Y.H., Qin Y., Tian M.M., Liu Y., Shen T. (2019). NSD2 circular RNA promotes metastasis of colorectal cancer by targeting miR-199b-5p-mediated DDR1 and JAG1 signalling. J. Pathol..

[76] Dai J., Zhuang Y., Tang M., Qian Q., Chen J. (2020). CircRNA UBAP2 facilitates the progression of colorectal cancer by regulating miR-199a/VEGFA pathway. Eur. Rev. Med. Pharm. Sci..

[77] Huang X.-Y., Zhang P.-F., Wei C.-Y., Peng R., Lu J.-C., Gao C., Cai J.-B., Yang X., Fan J., Ke A.-W. (2020). Circular RNA circMET drives immunosuppression and anti-PD1 therapy resistance in hepatocellular carcinoma via the miR-30-5p/snail/DPP4 axis. Mol. Cancer.

[78] Yao Z., Xu R., Yuan L., Xu M., Zhuang H., Li Y., Zhang Y., Lin N. (2019). Circ_0001955 facilitates hepatocellular carcinoma (HCC) tumorigenesis by sponging miR-516a-5p to release TRAF6 and MAPK11. Cell Death Dis..

[79] Liu Z., Yu Y., Huang Z., Kong Y., Hu X., Xiao W., Quan J., Fan X. (2019). CircRNA-5692 inhibits the progression of hepatocellular carcinoma by sponging miR-328-5p to enhance DAB2IP expression. Cell Death Dis..

[80] Zhong L., Wang Y., Cheng Y., Wang W., Lu B., Zhu L., Ma Y. (2018). Circular RNA circC3P1 suppresses hepatocellular carcinoma growth and metastasis through miR-4641/PCK1 pathway. Biochem. Biophys. Res. Commun..

[81] Wang Z., Zhao Y., Wang Y., Jin C. (2019). Circular RNA circHIAT1 inhibits cell growth in hepatocellular carcinoma by regulating miR-3171/PTEN axis. Biomed. Pharmacother..

[82] Figueiredo C., Camargo M.C., Leite M., Fuentes-Pananá E.M., Rabkin C.S., Machado J.C. (2017). Pathogenesis of gastric cancer: Genetics and molecular classification. Mol. Pathog. Signal Transduct. By Helicobacter Pylori.

[83] Sun H.-D., Xu Z.-P., Sun Z.-Q., Zhu B., Wang Q., Zhou J., Jin H., Zhao A., Tang W.-W., Cao X.-F. (2018). Down-regulation of circPVRL3 promotes the proliferation and migration of gastric cancer cells. Sci. Rep..

[84] Zhu Z., Rong Z., Luo Z., Yu Z., Zhang J., Qiu Z., Huang C. (2019). Circular RNA circNHSL1 promotes gastric cancer progression through the miR-1306-3p/SIX1/vimentin axis. Mol. Cancer.

[85] Luo Z., Rong Z., Zhang J., Zhu Z., Yu Z., Li T., Fu Z., Qiu Z., Huang C. (2020). Circular RNA circCCDC9 acts as a miR-6792-3p sponge to suppress the progression of gastric cancer through regulating CAV1 expression. Mol. Cancer.

[86] Seneviratne S., Lawrenson R., Scott N., Kim B., Shirley R., Campbell I. (2015). Breast cancer biology and ethnic disparities in breast cancer mortality in New Zealand: A cohort study. PLoS ONE.

[87] Huang F., Dang J., Zhang S., Cheng Z. (2020). Circular RNA hsa_circ_0008039 promotes proliferation, migration and invasion of breast cancer cells through upregulating CBX4 via sponging miR-515-5p. Eur. Rev. Med. Pharm. Sci..

[88] Liu Y., Lu C., Zhou Y., Zhang Z., Sun L. (2018). Circular RNA hsa_circ_0008039 promotes breast cancer cell proliferation and migration by regulating miR-432-5p/E2F3 axis. Biochem. Biophys. Res. Commun..

[89] Cao L., Wang M., Dong Y., Xu B., Chen J., Ding Y., Qiu S., Li L., Karamfilova Zaharieva E., Zhou X. (2020). Circular RNA circRNF20 promotes breast cancer tumorigenesis and Warburg effect through miR-487a/HIF-1α/HK2. Cell Death Dis..

[90] Fu B., Liu W., Zhu C., Li P., Wang L., Pan L., Li K., Cai P., Meng M., Wang Y. (2021). Circular RNA circBCBM1 promotes breast cancer brain metastasis by modulating miR-125a/BRD4 axis. Int. J. Biol. Sci..

[91] Li C., Liu H., Niu Q., Gao J. (2020). Circ_0000376, a novel circRNA, promotes the progression of non-small cell lung cancer through regulating the miR-1182/NOVA2 network. Cancer Manag. Res..

[92] Hu X., Wang P., Qu C., Zhang H., Li L. (2021). Circular RNA Circ_0000677 promotes cell proliferation by regulating microRNA-106b-5p/CCND1 in non-small cell lung cancer. Bioengineered.

[93] Qu D., Yan B., Xin R., Ma T. (2018). A novel circular RNA hsa_circ_0020123 exerts oncogenic properties through suppression of miR-144 in non-small cell lung cancer. Am. J. Cancer Res..

[94] Li L., Wan K., Xiong L., Liang S., Tou F., Guo S. (2020). CircRNA hsa_circ_0087862 acts as an oncogene in non-small cell lung cancer by targeting miR-1253/RAB3D axis. OncoTargets Ther..

[95] Han J., Zhao G., Ma X., Dong Q., Zhang H., Wang Y., Cui J. (2018). CircRNA circ-BANP-mediated miR-503/LARP1 signaling contributes to lung cancer progression. Biochem. Biophys. Res. Commun..

[96] Ma X., Yang X., Bao W., Li S., Liang S., Sun Y., Zhao Y., Wang J., Zhao C. (2018). Circular RNA circMAN2B2 facilitates lung cancer cell proliferation and invasion via miR-1275/FOXK1 axis. Biochem. Biophys. Res. Commun..

[97] Chi Y., Luo Q., Song Y., Yang F., Wang Y., Jin M., Zhang D. (2019). Circular RNA circPIP5K1A promotes non-small cell lung cancer proliferation and metastasis through miR-600/HIF-1α regulation. J. Cell. Biochem..

[98] Zhao Y., Zheng R., Chen J., Ning D. (2020). CircRNA CDR1as/miR-641/HOXA9 pathway regulated stemness contributes to cisplatin resistance in non-small cell lung cancer (NSCLC). Cancer Cell Int..

[99] Yang L., Wang J., Fan Y., Yu K., Jiao B., Su X. (2018). Hsa_circ_0046264 up-regulated BRCA2 to suppress lung cancer through targeting hsa-miR-1245. Respir. Res..

[100] Chen D., Ma W., Ke Z., Xie F. (2018). CircRNA hsa_circ_100395 regulates miR-1228/TCF21 pathway to inhibit lung cancer progression. Cell Cycle.

[101] Zhang Y., Zhao H., Zhang L. (2018). Identification of the tumor-suppressive function of circular RNA FOXO3 in non-small cell lung cancer through sponging miR-155. Mol. Med. Rep..

[102] Chen L.-Y., Wang L., Ren Y.-X., Pang Z., Liu Y., Sun X.-D., Tu J., Zhi Z., Qin Y., Sun L.-N. (2020). The circular RNA circ-ERBIN promotes growth and metastasis of colorectal cancer by miR-125a-5p and miR-138-5p/4EBP-1 mediated cap-independent HIF-1α translation. Mol. Cancer.

[103] Chen H., Wu C., Luo L., Wang Y., Peng F. (2021). circ_0000467 promotes the proliferation, metastasis, and angiogenesis in colorectal cancer cells through regulating KLF12 expression by sponging miR-4766-5p. Open Med..

[104] Deng Z., Li X., Wang H., Geng Y., Cai Y., Tang Y., Wang Y., Yu X., Li L., Li R. (2020). Dysregulation of CircRNA_0001946 contributes to the proliferation and metastasis of colorectal cancer cells by targeting MicroRNA-135a-5p. Front. Genet..

[105] Yang L., Sun H., Liu X., Chen J., Tian Z., Xu J., Xiang B., Qin B. (2020). Circular RNA hsa_circ_0004277 contributes to malignant phenotype of colorectal cancer by sponging miR-512-5p to upregulate the expression of PTMA. J. Cell. Physiol..

[106] Wang X., Zhang H., Yang H., Bai M., Ning T., Deng T., Liu R., Fan Q., Zhu K., Li J. (2020). Exosome-delivered circRNA promotes glycolysis to induce chemoresistance through the miR-122-PKM2 axis in colorectal cancer. Mol. Oncol..

[107] He J.H., Han Z.P., Luo J.G., Jiang J.W., Zhou J.B., Chen W.M., Lv Y.B., He M.L., Zheng L., Li Y.G. (2020). Hsa_Circ_0007843 acts as a mIR-518c-5p sponge to regulate the migration and invasion of colon cancer SW480 cells. Front. Genet..

[108] Xiao H., Liu M. (2020). Circular RNA hsa_circ_0053277 promotes the development of colorectal cancer by upregulating matrix metallopeptidase 14 via miR-2467-3p sequestration. J. Cell. Physiol..

[109] Wang X., Ren Y., Ma S., Wang S. (2020). Circular RNA 0060745, a novel circRNA, promotes colorectal cancer cell proliferation and metastasis through miR-4736 sponging. OncoTargets Ther..

[110] Chen P., Yao Y., Yang N., Gong L., Kong Y., Wu A. (2020). Circular RNA circCTNNA1 promotes colorectal cancer progression by sponging miR-149-5p and regulating FOXM1 expression. Cell Death Dis..

[111] Zeng K., Chen X., Xu M., Liu X., Hu X., Xu T., Sun H., Pan Y., He B., Wang S. (2018). CircHIPK3 promotes colorectal cancer growth and metastasis by sponging miR-7. Cell Death Dis..

[112] Yang B., Du K., Yang C., Xiang L., Xu Y., Cao C., Zhang J., Liu W. (2020). CircPRMT5 circular RNA promotes proliferation of colorectal cancer through sponging miR-377 to induce E2F3 expression. J. Cell. Mol. Med..

[113] He J.-H., Li Y.-G., Han Z.-P., Zhou J.-B., Chen W.-M., Lv Y.-B., He M.-L., Zuo J.-D., Zheng L. (2018). The CircRNA-ACAP2/Hsa-miR-21-5p/Tiam1 regulatory feedback circuit affects the proliferation, migration, and invasion of colon cancer SW480 cells. Cell. Physiol. Biochem..

[114] Wang Y., Gao R., Li J., Tang S., Li S., Tong Q., Mao Y. (2020). Circular RNA hsa_circ_0003141 promotes tumorigenesis of hepatocellular carcinoma via a miR-1827/UBAP2 axis. Aging.

[115] Yu Y., Bian L., Liu R., Wang Y., Xiao X. (2021). Circular RNA hsa_circ_0061395 accelerates hepatocellular carcinoma progression via regulation of the miR-877-5p/PIK3R3 axis. Cancer Cell Int..

[116] Bai N., Peng E., Qiu X., Lyu N., Zhang Z., Tao Y., Li X., Wang Z. (2018). circFBLIM1 act as a ceRNA to promote hepatocellular cancer progression by sponging miR-346. J. Exp. Clin. Cancer Res..

[117] Yu X., Sheng P., Sun J., Zhao X., Zhang J., Li Y., Zhang Y., Zhang W., Wang J., Liu K. (2020). The circular RNA circMAST1 promotes hepatocellular carcinoma cell proliferation and migration by sponging miR-1299 and regulating CTNND1 expression. Cell Death Dis..

[118] Li S., Weng J., Song F., Li L., Xiao C., Yang W., Xu J. (2020). Circular RNA circZNF566 promotes hepatocellular carcinoma progression by sponging miR-4738-3p and regulating TDO2 expression. Cell Death Dis..

[119] Jiang W., Wen D., Gong L., Wang Y., Liu Z., Yin F. (2018). Circular RNA hsa_circ_0000673 promotes hepatocellular carcinoma malignance by decreasing miR-767-3p targeting SET. Biochem. Biophys. Res. Commun..

[120] Guan Z., Tan J., Gao W., Li X., Yang Y., Li X., Li Y., Wang Q. (2019). Circular RNA hsa_circ_0016788 regulates hepatocellular carcinoma tumorigenesis through miR-486/CDK4 pathway. J. Cell. Physiol..

[121] Zhu Q., Lu G., Luo Z., Gui F., Wu J., Zhang D., Ni Y. (2018). CircRNA circ_0067934 promotes tumor growth and metastasis in hepatocellular carcinoma through regulation of miR-1324/FZD5/Wnt/β-catenin axis. Biochem. Biophys. Res. Commun..

[122] Zhan W., Liao X., Chen Z., Li L., Tian T., Yu L., Wang W., Hu Q. (2020). Circular RNA hsa_circRNA_103809 promoted hepatocellular carcinoma development by regulating miR-377-3p/FGFR1/ERK axis. J. Cell. Physiol..

[123] Huang G., Liang M., Liu H., Huang J., Li P., Wang C., Zhang Y., Lin Y., Jiang X. (2020). CircRNA hsa_circRNA_104348 promotes hepatocellular carcinoma progression through modulating miR-187-3p/RTKN2 axis and activating Wnt/β-catenin pathway. Cell Death Dis..

[124] Lv S., Li Y., Ning H., Zhang M., Jia Q., Wang X. (2021). CircRNA GFRA1 promotes hepatocellular carcinoma progression by modulating the miR-498/NAP1L3 axis. Sci. Rep..

[125] Chen G., Shi Y., Liu M., Sun J. (2018). circHIPK3 regulates cell proliferation and migration by sponging miR-124 and regulating AQP3 expression in hepatocellular carcinoma. Cell Death Dis..

[126] Li Q., Pan X., Zhu D., Deng Z., Jiang R., Wang X. (2019). Circular RNA MAT2B promotes glycolysis and malignancy of hepatocellular carcinoma through the miR-338-3p/PKM2 axis under hypoxic stress. Hepatology.

[127] He Y., Wang Y., Liu L., Liu S., Liang L., Chen Y., Zhu Z. (2020). Circular RNA circ_0006282 contributes to the progression of gastric cancer by sponging miR-155 to upregulate the expression of FBXO22. OncoTargets Ther..

[128] Huang S., Zhang X., Guan B., Sun P., Hong C.T., Peng J., Tang S., Yang J. (2019). A novel circular RNA hsa_circ_0008035 contributes to gastric cancer tumorigenesis through targeting the miR-375/YBX1 axis. Am. J. Transl. Res..

[129] Huang X., Li Z., Zhang Q., Wang W., Li B., Wang L., Xu Z., Zeng A., Zhang X., Zhang X. (2019). Circular RNA AKT3 upregulates PIK3R1 to enhance cisplatin resistance in gastric cancer via miR-198 suppression. Mol. Cancer.

[130] Zhang L., Song X., Chen X., Wang Q., Zheng X., Wu C., Jiang J. (2019). Circular RNA CircCACTIN promotes gastric cancer progression by sponging MiR-331-3p and regulating TGFBR1 expression. Int. J. Biol. Sci..

[131] Chen D.-L., Sheng H., Zhang D.-S., Jin Y., Zhao B.-T., Chen N., Song K., Xu R.-H. (2021). The circular RNA circDLG1 promotes gastric cancer progression and anti-PD-1 resistance through the regulation of CXCL12 by sponging miR-141-3p. Mol. Cancer.

[132] Zhang X., Wang S., Wang H., Cao J., Huang X., Chen Z., Xu P., Sun G., Xu J., Lv J. (2019). Circular RNA circNRIP1 acts as a microRNA-149-5p sponge to promote gastric cancer progression via the AKT1/mTOR pathway. Mol. Cancer.

[133] Ouyang Y., Li Y., Huang Y., Li X., Zhu Y., Long Y., Wang Y., Guo X., Gong K. (2019). CircRNA circPDSS1 promotes the gastric cancer progression by sponging miR-186-5p and modulating NEK2. J. Cell. Physiol..

[134] Fan D., Wang C., Wang D., Zhang N., Yi T. (2021). Circular RNA circ_0000039 enhances gastric cancer progression through miR-1292-5p/DEK axis. Cancer Biomark..

[135] Lu J., Wang Y.-h., Yoon C., Huang X.-y., Xu Y., Xie J.-w., Wang J.-b., Lin J.-x., Chen Q.-y., Cao L.-l. (2020). Circular RNA circ-RanGAP1 regulates VEGFA expression by targeting miR-877–3p to facilitate gastric cancer invasion and metastasis. Cancer Lett..

[136] Pan H., Li T., Jiang Y., Pan C., Ding Y., Huang Z., Yu H., Kong D. (2018). Overexpression of circular RNA ciRS-7 abrogates the tumor suppressive effect of miR-7 on gastric cancer via PTEN/PI3K/AKT signaling pathway. J. Cell. Biochem..

[137] Lv L., Du J., Wang D., Yan Z. (2022). Circular RNA hsa_circ_0026344 suppresses gastric cancer cell proliferation, migration and invasion via the miR-590-5p/PDCD4 axis. J. Pharm. Pharmacol..

[138] Zhang J., Liu H., Hou L., Wang G., Zhang R., Huang Y., Chen X., Zhu J. (2017). Circular RNA_LARP4 inhibits cell proliferation and invasion of gastric cancer by sponging miR-424-5p and regulating LATS1 expression. Mol. Cancer.

[139] Sun G., Li Z., He Z., Wang W., Wang S., Zhang X., Cao J., Xu P., Wang H., Huang X. (2020). Circular RNA MCTP2 inhibits cisplatin resistance in gastric cancer by miR-99a-5p-mediated induction of MTMR3 expression. J. Exp. Clin. Cancer Res..

[140] Qiu Z., Wang L., Liu H. (2021). Hsa_circ_0001982 promotes the progression of breast cancer through miR-1287-5p/MUC19 axis under hypoxia. World J. Surg. Oncol..

[141] Xu Y., Qian C., Liu C., Fu Y., Zhu K., Niu Z., Liu J. (2022). Investigation of the Mechanism of hsa_circ_000 1429 Adsorbed miR-205 to Regulate KDM4A and Promote Breast Cancer Metastasis. Contrast Media Mol. Imaging.

[142] Xu Y., Yao Y., Leng K., Ji D., Qu L., Liu Y., Cui Y. (2018). Increased expression of circular RNA circ_0005230 indicates dismal prognosis in breast cancer and regulates cell proliferation and invasion via miR-618/CBX8 signal pathway. Cell. Physiol. Biochem..

[143] Liu L.H., Tian Q.Q., Liu J., Zhou Y., Yong H. (2019). Upregulation of hsa_circ_0136666 contributes to breast cancer progression by sponging miR-1299 and targeting CDK6. J. Cell. Biochem..

[144] Yao Y., Fang Z. (2019). Circular RNA-100219 promotes breast cancer progression by binding to microRNA-485-3p. J BUON.

[145] Zeng K., He B., Yang B.B., Xu T., Chen X., Xu M., Liu X., Sun H., Pan Y., Wang S. (2018). The pro-metastasis effect of circANKS1B in breast cancer. Mol. Cancer.

[146] Qu Y., Dou P., Hu M., Xu J., Xia W., Sun H. (2019). circRNA-CER mediates malignant progression of breast cancer through targeting the miR-136/MMP13 axis. Mol. Med. Rep..

[147] Zhang W., Liu H., Jiang J., Yang Y., Wang W., Jia Z. (2021). CircRNA circFOXK2 facilitates oncogenesis in breast cancer via IGF2BP3/miR-370 axis. Aging.

[148] Wang N., Gu Y., Li L., Wang F., Lv P., Xiong Y., Qiu X. (2018). Circular RNA circMYO9B facilitates breast cancer cell proliferation and invasiveness via upregulating FOXP4 expression by sponging miR-4316. Arch. Biochem. Biophys..

[149] Lin G., Wang S., Zhang X., Wang D. (2020). Circular RNA circPLK1 promotes breast cancer cell proliferation, migration and invasion by regulating miR-4500/IGF1 axis. Cancer Cell Int..

[150] Zhang H., Ge Z., Wang Z., Gao Y., Wang Y., Qu X. (2021). Circular RNA RHOT1 promotes progression and inhibits ferroptosis via mir-106a-5p/STAT3 axis in breast cancer. Aging.

[151] Zhou Y., Liu X., Lan J., Wan Y., Zhu X. (2020). Circular RNA circRPPH1 promotes triple-negative breast cancer progression via the miR-556-5p/YAP1 axis. Am. J. Transl. Res..

[152] Chen Z.G., Zhao H.J., Lin L., Liu J.B., Bai J.Z., Wang G.S. (2020). Circular RNA CirCHIPK3 promotes cell proliferation and invasion of breast cancer by sponging miR-193a/HMGB1/PI3K/AKT axis. Thorac. Cancer.

[153] Zhang X.-Y., Mao L. (2021). Circular RNA Circ_0000442 acts as a sponge of MiR-148b-3p to suppress breast cancer via PTEN/PI3K/Akt signaling pathway. Gene.

[154] Meng L., Chang S., Sang Y., Ding P., Wang L., Nan X., Xu R., Liu F., Gu L., Zheng Y. (2022). Circular RNA circCCDC85A inhibits breast cancer progression via acting as a miR-550a-5p sponge to enhance MOB1A expression. Breast Cancer Res..

[155] Liu W.-L., Wang H.-x., Shi C.-x., Shi F.-y., Zhao L.-y., Zhao W., Wang G.-h. (2019). MicroRNA-1269 promotes cell proliferation via the AKT signaling pathway by targeting RASSF9 in human gastric cancer. Cancer Cell Int..

[156] Wu F., Liu F., Dong L., Yang H., He X., Li L., Zhao L., Jin S., Li G. (2018). miR-1273g silences MAGEA3/6 to inhibit human colorectal cancer cell growth via activation of AMPK signaling. Cancer Lett..

[157] Yu W., Jiang H., Zhang H., Li J. (2018). Hsa_circ_0003998 promotes cell proliferation and invasion by targeting miR-326 in non-small cell lung cancer. OncoTargets Ther..

[158] Fan Z., Bai Y., Zhang Q., Qian P. (2020). CircRNA circ_POLA2 promotes lung cancer cell stemness via regulating the miR-326/GNB1 axis. Environ. Toxicol..

[159] He S., Yang J., Jiang S., Li Y., Han X. (2020). Circular RNA circ_0000517 regulates hepatocellular carcinoma development via miR-326/IGF1R axis. Cancer Cell Int..

[160] Pan G., Mao A., Liu J., Lu J., Ding J., Liu W. (2020). Circular RNA hsa_circ_0061825 (circ-TFF1) contributes to breast cancer progression through targeting miR-326/TFF1 signalling. Cell Prolif..

[161] Wang S., Hu Y., Lv X., Li B., Gu D., Li Y., Sun Y., Su Y. (2019). Circ-0000284 arouses malignant phenotype of cholangiocarcinoma cells and regulates the biological functions of peripheral cells through cellular communication. Clin. Sci..

[162] Yang D., Hu Z., Zhang Y., Zhang X., Xu J., Fu H., Zhu Z., Feng D., Cai Q. (2021). CircHIPK3 promotes the tumorigenesis and development of gastric cancer through miR-637/AKT1 pathway. Front. Oncol..

[163] Wen Y., Li B., He M., Teng S., Sun Y., Wang G. (2021). circHIPK3 promotes proliferation and migration and invasion via regulation of miR-637/HDAC4 signaling in osteosarcoma cells. Oncol. Rep..

[164] Xu L., Feng X., Hao X., Wang P., Zhang Y., Zheng X., Li L., Ren S., Zhang M., Xu M. (2019). CircSETD3 (Hsa_circ_0000567) acts as a sponge for microRNA-421 inhibiting hepatocellular carcinoma growth. J. Exp. Clin. Cancer Res..

[165] Xiao W., Zheng S., Zou Y., Yang A., Xie X., Tang H., Xie X. (2019). CircAHNAK1 inhibits proliferation and metastasis of triple-negative breast cancer by modulating miR-421 and RASA1. Aging.

[166] Yang C., Yuan W., Yang X., Li P., Wang J., Han J., Tao J., Li P., Yang H., Lv Q. (2018). Circular RNA circ-ITCH inhibits bladder cancer progression by sponging miR-17/miR-224 and regulating p21, PTEN expression. Mol. Cancer.

[167] Li P., Chen H., Chen S., Mo X., Li T., Xiao B., Yu R., Guo J. (2017). Circular RNA 0000096 affects cell growth and migration in gastric cancer. Br. J. Cancer.

[168] Wang Y., Yin H., Chen X. (2021). Circ-LDLRAD3 enhances cell growth, migration, and invasion and inhibits apoptosis by regulating MiR-224-5p/NRP2 axis in gastric cancer. Dig. Dis. Sci..

[169] Chen T., Yang Z., Liu C., Wang L., Yang J., Chen L., Li W. (2019). Circ_0078767 suppresses non-small-cell lung cancer by protecting RASSF1A expression via sponging miR-330-3p. Cell Prolif..

[170] Wang Y., Xu R., Zhang D., Lu T., Yu W., Wo Y., Liu A., Sui T., Cui J., Qin Y. (2019). Circ-ZKSCAN1 regulates FAM83A expression and inactivates MAPK signaling by targeting miR-330-5p to promote non-small cell lung cancer progression. Transl. Lung Cancer Res..

[171] Lu C., Fu L., Qian X., Dou L., Cang S. (2020). Knockdown of circular RNA circ-FARSA restricts colorectal cancer cell growth through regulation of miR-330-5p/LASP1 axis. Arch. Biochem. Biophys..

[172] Fang C., Huang X., Dai J., He W., Xu L., Sun F. (2022). The circular RNA circFARSA sponges microRNA-330-5p in tumor cells with bladder cancer phenotype. BMC Cancer.

[173] Jia Q., Ye L., Xu S., Xiao H., Xu S., Shi Z., Li J., Chen Z. (2020). Circular RNA 0007255 regulates the progression of breast cancer through miR-335-5p/SIX2 axis. Thorac. Cancer.

[174] Liang H.-F., Zhang X.-Z., Liu B.-G., Jia G.-T., Li W.-L. (2017). Circular RNA circ-ABCB10 promotes breast cancer proliferation and progression through sponging miR-1271. Am. J. Cancer Res..

[175] Hu C., Wang Y., Li A., Zhang J., Xue F., Zhu L. (2019). Overexpressed circ_0067934 acts as an oncogene to facilitate cervical cancer progression via the miR-545/EIF3C axis. J. Cell. Physiol..

[176] Liu J., Wang D., Long Z., Liu J., Li W. (2018). CircRNA8924 promotes cervical cancer cell proliferation, migration and invasion by competitively binding to MiR-518d-5p/519-5p family and modulating the expression of CBX8. Cell. Physiol. Biochem..

[177] Xia L., Wu L., Bao J., Li Q., Chen X., Xia H., Xia R. (2018). Circular RNA circ-CBFB promotes proliferation and inhibits apoptosis in chronic lymphocytic leukemia through regulating miR-607/FZD3/Wnt/β-catenin pathway. Biochem. Biophys. Res. Commun..

[178] Zhang Q., Zhang C., Ma J.-X., Ren H., Sun Y., Xu J.-Z. (2019). Circular RNA PIP5K1A promotes colon cancer development through inhibiting miR-1273a. World J. Gastroenterol..

[179] Lin C., Ma M., Zhang Y., Li L., Long F., Xie C., Xiao H., Liu T., Tian B., Yang K. (2022). The N6-methyladenosine modification of circALG1 promotes the metastasis of colorectal cancer mediated by the miR-342-5p/PGF signalling pathway. Mol. Cancer.

[180] Li R., Wu B., Xia J., Ye L., Yang X. (2019). Circular RNA hsa_circRNA_102958 promotes tumorigenesis of colorectal cancer via miR-585/CDC25B axis. Cancer Manag. Res..

[181] Ma Z., Ma J., Lang B., Xu F., Zhang B., Wang X. (2022). Circ_0001982 Up-regulates the Expression of E2F1 by Adsorbing miR-1205 to Facilitate the Progression of Glioma. Mol. Biotechnol..

[182] Yi C., Li H., Li D., Qin X., Wang J., Liu Y., Liu Z., Zhang J. (2019). Upregulation of circular RNA circ_0034642 indicates unfavorable prognosis in glioma and facilitates cell proliferation and invasion via the miR-1205/BATF3 axis. J. Cell. Biochem..

[183] Qin L., Zhan Z., Wei C., Li X., Zhang T., Li J. (2021). Hsa-circRNA-G004213 promotes cisplatin sensitivity by regulating miR-513b-5p/PRPF39 in liver cancer Corrigendum in/10.3892/mmr. 2021.12359. Mol. Med. Rep..

[184] Liu Q., Wang C., Jiang Z., Li S., Li F., Tan H.B., Yue S.Y. (2020). circRNA 001306 enhances hepatocellular carcinoma growth by up-regulating CDK16 expression via sponging miR-584-5p. J. Cell. Mol. Med..

[185] Zhang X., Xu Y., Qian Z., Zheng W., Wu Q., Chen Y., Zhu G., Liu Y., Bian Z., Xu W. (2018). circRNA_104075 stimulates YAP-dependent tumorigenesis through the regulation of HNF4a and may serve as a diagnostic marker in hepatocellular carcinoma. Cell Death Dis..

[186] Liu G., Shi H., Deng L., Zheng H., Kong W., Wen X., Bi H. (2019). Circular RNA circ-FOXM1 facilitates cell progression as ceRNA to target PPDPF and MACC1 by sponging miR-1304-5p in non-small cell lung cancer. Biochem. Biophys. Res. Commun..

[187] Zhou Y., Zheng X., Xu B., Chen L., Wang Q., Deng H., Jiang J. (2019). Circular RNA hsa_circ_0004015 regulates the proliferation, invasion, and TKI drug resistance of non-small cell lung cancer by miR-1183/PDPK1 signaling pathway. Biochem. Biophys. Res. Commun..

[188] Zhang S., Zeng X., Ding T., Guo L., Li Y., Ou S., Yuan H. (2018). Microarray profile of circular RNAs identifies hsa_circ_0014130 as a new circular RNA biomarker in non-small cell lung cancer. Sci. Rep..

[189] Wei H., Pan L., Tao D., Li R. (2018). Circular RNA circZFR contributes to papillary thyroid cancer cell proliferation and invasion by sponging miR-1261 and facilitating C8orf4 expression. Biochem. Biophys. Res. Commun..

[190] Li B., Liang L., Chen Y., Liu J., Wang Z., Mao Y., Zhao K., Chen J. (2022). Circ_0008287 promotes immune escape of gastric cancer cells through impairing microRNA-548c-3p-dependent inhibition of CLIC1. Int. Immunopharmacol..

[191] Li Y., Zheng F., Xiao X., Xie F., Tao D., Huang C., Liu D., Wang M., Wang L., Zeng F. (2017). Circ HIPK 3 sponges miR-558 to suppress heparanase expression in bladder cancer cells. EMBO Rep..

[192] Yang S., Gao S., Liu T., Liu J., Zheng X., Li Z. (2021). Circular RNA SMARCA5 functions as an anti-tumor candidate in colon cancer by sponging microRNA-552. Cell Cycle.

[193] Gao S., Yu Y., Liu L., Meng J., Li G. (2019). Circular RNA hsa_circ_0007059 restrains proliferation and epithelial-mesenchymal transition in lung cancer cells via inhibiting microRNA-378. Life Sci..

[194] Wu Z., Shi W., Jiang C. (2018). Overexpressing circular RNA hsa_circ_0002052 impairs osteosarcoma progression via inhibiting Wnt/β-catenin pathway by regulating miR-1205/APC2 axis. Biochem. Biophys. Res. Commun..

[195] Fang J., Hong H., Xue X., Zhu X., Jiang L., Qin M., Liang H., Gao L. (2019). A novel circular RNA, circFAT1 (e2), inhibits gastric cancer progression by targeting miR-548g in the cytoplasm and interacting with YBX1 in the nucleus. Cancer Lett..

