# Correction: Kim et al. The Tumorigenic Role of Circular RNA-MicroRNA Axis in Cancer. *Int. J. Mol. Sci.* 2023, *24*, 3050

**DOI:** 10.3390/ijms27167477

**Published:** 2026-08-21

**Authors:** Woo Ryung Kim, Eun Gyung Park, Du Hyeong Lee, Yun Ju Lee, Woo Hyeon Bae, Heui-Soo Kim

**Affiliations:** 1Department of Integrated Biological Sciences, Pusan National University, Busan 46241, Republic of Korea; dnfud647@pusan.ac.kr (W.R.K.); ehdtodt@pusan.ac.kr (E.G.P.); doo2080@naver.com (D.H.L.); lsg5821@naver.com (Y.J.L.); qodngus96@naver.com (W.H.B.); 2Institute of Systems Biology, Pusan National University, Busan 46241, Republic of Korea; 3Department of Biological Sciences, College of Natural Sciences, Pusan National University, Busan 46241, Republic of Korea

## References

After the publication of this review article [1], references 69, 85, 100, 116, 119, 129, 133, 139, 148, 166, 200, and 207 in the original manuscript were retracted by the publisher. Upon recognition of this issue, the affected references were carefully reviewed and subsequently removed from the reference list.

## Text Correction

The following revisions were made to remove the retracted references and the corresponding content from the manuscript:

In Section 2.2 Colon Cancer, the content corresponding to reference [69] was removed (lines 9–11).

In Section 2.4 Gastric Cancer, the content corresponding to reference [85] was removed (lines 9–11).

In Section 3 MDTEs Regulated by Oncogenic CircRNAs and Tumor-Suppressive circRNAs in Various Cancers, the content corresponding to reference [166] was removed (lines 9–12).

In Table 1, the content corresponding to references [69, 85, 100, 116, 119, 129, 133, 139, and 148] was removed.

In Table 2, the content corresponding to references [200 and 207] was removed.

69. Deng, Q.; Wang, C.; Hao, R.; Yang, Q. Circ_0001982 accelerates the progression of colorectal cancer via sponging microRNA-144. *Eur. Rev. Med. Pharm. Sci.* **2020**, *24*, 1755–1762.

85. Yu, Z.; Lan, J.; Li, W.; Jin, L.; Qi, F.; Yu, C.; Zhu, H. Circular RNA hsa_circ_0002360 promotes proliferation and invasion and inhibits oxidative stress in gastric cancer by sponging miR-629-3p and regulating the PDLIM4 expression. *Oxidative Med. Cell. Longev.* **2022**, *2022*, 2775433.

100. Li, X.; Zhang, Z.; Jiang, H.; Li, Q.; Wang, R.; Pan, H.; Niu, Y.; Liu, F.; Gu, H.; Fan, X. Circular RNA circPVT1 promotes proliferation and invasion through sponging miR-125b and activating E2F2 signaling in non-small cell lung cancer. *Cell. Physiol. Biochem.* **2018**, *51*, 2324–2340.

116. Jiang, Z.; Hou, Z.; Liu, W.; Yu, Z.; Liang, Z.; Chen, S. Circular RNA protein tyrosine kinase 2 (circPTK2) promotes colorectal cancer proliferation, migration, invasion and chemoresistance. *Bioengineered* **2022**, *13*, 810–823.

119. Li, Z.; Liu, Y.; Yan, J.; Zeng, Q.; Hu, Y.; Wang, H.; Li, H.; Li, J.; Yu, Z. Circular RNA hsa_circ_0056836 functions an oncogenic gene in hepatocellular carcinoma through modulating miR-766-3p/FOSL2 axis. *Aging* **2020**, *12*, 2485.

129. Yu, L.; Gong, X.; Sun, L.; Zhou, Q.; Lu, B.; Zhu, L. The circular RNA Cdr1as act as an oncogene in hepatocellular carcinoma through targeting miR-7 expression. *PLoS ONE* **2016**, *11*, e0158347.

133. Zhang, H.; Zhu, L.; Bai, M.; Liu, Y.; Zhan, Y.; Deng, T.; Yang, H.; Sun, W.; Wang, X.; Zhu, K. Exosomal circRNA derived from gastric tumor promotes white adipose browning by targeting the miR-133/PRDM16 pathway. *Int. J. Cancer* **2019**, *144*, 2501–2515.

139. Song, R.; Li, Y.; Hao, W.; Yang, L.; Chen, B.; Zhao, Y.; Sun, B.; Xu, F. Circular RNA MTO1 inhibits gastric cancer progression by elevating PAWR via sponging miR-199a-3p. *Cell Cycle* **2020**, *19*, 3127–3139.

148. Rong, D.; Lu, C.; Zhang, B.; Fu, K.; Zhao, S.; Tang, W.; Cao, H. CircPSMC3 suppresses the proliferation and metastasis of gastric cancer by acting as a competitive endogenous RNA through sponging miR-296-5p. *Mol. Cancer* **2019**, *18*, 25.

166. Xu, X.; Cao, L.; Zhang, Y.; Lian, H.; Sun, Z.; Cui, Y. MicroRNA-1246 inhibits cell invasion and epithelial mesenchymal transition process by targeting CXCR4 in lung cancer cells. *Cancer Biomark*. **2018**, *21*, 251–260.

200. Zhang, Z.; Wu, H.; Chen, Z.; Li, G.; Liu, B. Circular RNA ATXN7 promotes the development of gastric cancer through sponging miR-4319 and regulating ENTPD4. *Cancer Cell Int.* **2020**, *20*, 25.

207. Liu, T.; Liu, S.; Xu, Y.; Shu, R.; Wang, F.; Chen, C.; Zeng, Y.; Luo, H. Circular RNA-ZFR inhibited cell proliferation and promoted apoptosis in gastric cancer by sponging miR-130a/miR-107 and modulating PTEN. *Cancer Res. Treat. Off. J. Korean Cancer Assoc.* **2018**, *50*, 1396–1417. 

With this correction, the order of some references has been adjusted accordingly.

The retracted articles corresponded to only a subset of the cited examples; they do not affect the scientific conclusions of the study. This correction was approved by the Academic Editor. The original publication has also been updated.
